# The inflammaging microenvironment induces dysfunctional rewiring of Tfh cell differentiation

**DOI:** 10.1172/jci.insight.187271

**Published:** 2025-03-04

**Authors:** Cody S. Nelson, Manuel A. Podestà, Maya G. Gempler, Jeong-Mi Lee, Cole J. Batty, Peterson G. Mathenge, Asra Sainju, Matthew R. Chang, Hanzhong Ke, Pragya Chandrakar, Elsa Bechu, Sierra Richardson, Cecilia B. Cavazzoni, Stefan G. Tullius, Reza Abdi, Musie Ghebremichael, Marcia C. Haigis, Wayne A. Marasco, Peter T. Sage

**Affiliations:** 1 Transplantation Research Center, Division of Renal Medicine, Department of Medicine; and; 2 Division of Allergy and Clinical Immunology, Department of Medicine, Brigham and Women’s Hospital, Boston, Massachusetts, USA.; 3 Unit of Nephrology, Dialysis, and Renal Transplantation, Fondazione IRCCS Ca’ Granda Ospedale Maggiore Policlinico, Milan, Italy.; 4 Department of Cancer Immunology and Virology, Dana Farber Cancer Institute, Boston, Massachusetts, USA.; 5 Division of Transplant Surgery, Department of Surgery, Brigham and Women’s Hospital, Harvard Medical School, Boston, Massachusetts, USA.; 6 Ragon Institute of Mass General Brigham, MIT, and Harvard, Cambridge, Massachusetts, USA.; 7 Department of Cell Biology, Blavatnik Institute, Harvard Medical School, Boston, Massachusetts, USA.

**Keywords:** Aging, Immunology, Adaptive immunity, Influenza, T cells

## Abstract

Humoral immunity is orchestrated by follicular helper T (Tfh) cells, which promote cognate B cells to produce high-affinity, protective antibodies. In aged individuals, humoral immunity after vaccination is diminished despite the presence of Tfh cells, suggesting defects after initial Tfh cell formation. In this study, we utilized both murine and human systems to investigate how aging alters Tfh cell differentiation after influenza vaccination. We found that young Tfh cells underwent progressive differentiation after influenza vaccination, culminating in clonal expansion of effector-like cells in both draining lymph nodes and blood. In aging, early stages of Tfh cell development occurred normally. However, aging rewired the later stages of development in Tfh cells, resulting in a transcriptional program reflective of cellular senescence, sustained pro-inflammatory cytokine production, and metabolic reprogramming. We investigated the extent to which this rewiring of aged Tfh cells is due to the age-associated inflammatory (“inflammaging”) microenvironment and found that this setting was sufficient to both block the transition of Tfh cells to a post-effector resting state and skew Tfh cells toward the age-rewired state. Together, these data suggest that aging dampens humoral immunity by cytokine-mediated rewiring of late effector Tfh cell differentiation into an activated, yet less functional, cellular state.

## Abstract 2

Nelson et al. demonstrate that a factor limiting immunity in older individuals is an inflammatory microenvironment that redirects later stages of Tfh differentiation to a dysfunctional state.

## Graphical abstract

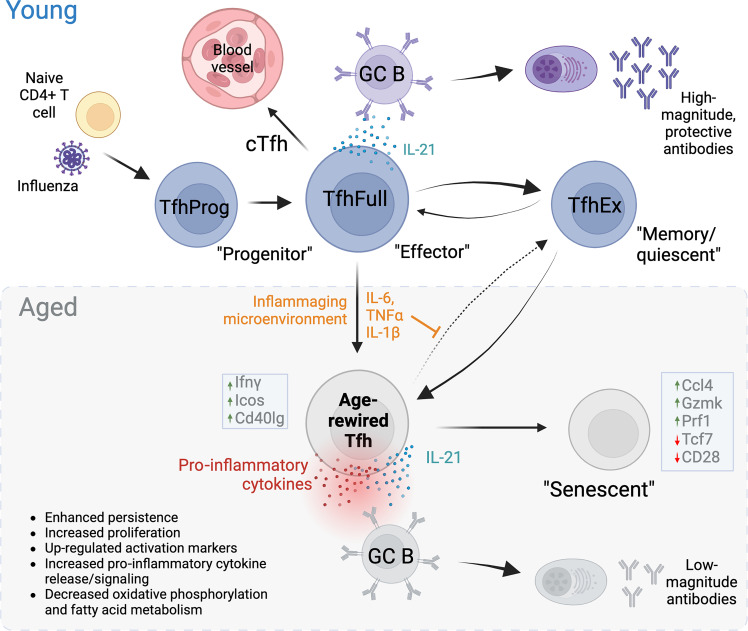

## Introduction

The average age of the US population continues to rise, with approximately 1 in 5 US citizens currently exceeding 65 years of age. It is established that the adaptive immune system changes with age and frailty. While there is a lack of consensus regarding the relationship between age and vaccine responses, some studies suggest that the magnitude of protective antibodies elicited by both primary vaccination and vaccine boosting (e.g., secondary/repeat vaccination) is attenuated in aged individuals (1–5). In addition, aged individuals exhibit a reduced durability of vaccine-elicited antibodies, with titers that wane more rapidly than those in young individuals (6, 7). There is increasing recognition that aged lymphocytes possess unique cellular programming and are not simply attenuated versions of their youthful counterparts. The aging microenvironment contains increased concentrations of some pro-inflammatory cytokines produced by both immune and stromal cells, which creates a distinctive “inflammaging” microenvironment (8–10). However, the precise mechanisms by which aging alters antibody-mediated immunity after vaccination are poorly understood because of the complexity of the aging process.

The goal of vaccination is to elicit protective antibodies that persist for long periods; however, in some circumstances, such as influenza vaccination, protection wanes rapidly (11). T follicular helper (Tfh) cells stimulate B cells to undergo affinity maturation in germinal centers (GCs), eventually culminating in the development of memory B cells that respond upon antigen reexposure or plasma cells that produce high-affinity antibodies (12, 13). Newer data suggest that Tfh cells undergo progressive differentiation, a process that is required to generate effector Tfh cells that promote the GC reaction (14). Some effector Tfh cells can partially downregulate the effector program but remain epigenetically poised, called a TfhEx state. Furthermore, a subset of Tfh cells assume a memory-like phenotype, exit the GC, and can be found circulating in the peripheral blood (15). Since the magnitude of circulating Tfh (cTfh) cells correlates with the quality, quantity, and longevity of antibody responses in some settings, a fundamental goal of vaccination is to induce sustained cTfh responses (16–18). Conversely, T follicular regulatory (Tfr) cells dampen Tfh-mediated B cell activation and antibody production, while optimizing affinity maturation (19).

The current paradigm suggests the GC reaction is altered in aged individuals. B cells exhibit age-related functional changes, including decreased class switch recombination, bimodal somatic hypermutation, decreased plasma cell differentiation, and enhanced expression of the transcription factor T-bet (19, 20). Furthermore, aged Tfh cells exhibit altered tissue localization as well as transcriptional programming (21, 22). Intriguingly, the total frequency of Tfh cells is higher in aged individuals (23), which is accompanied by an increase in the prototypical Tfh cytokine IL-21 in the serum of some aged individuals (24). Therefore, aging does not limit the presence of Tfh cells, per se, but may regulate the tissue localization and transcriptional profile of Tfh cells. However, how the complex physiology of aging alters Tfh differentiation is poorly understood.

In this study, we assessed how aging alters Tfh differentiation in response to seasonal influenza vaccination, utilizing both murine models and human clinical samples. In both mice and humans, Tfh cells underwent progressive Tfh development in response to influenza vaccination. Settings of aging were able to support normal early stages of Tfh development in lymph nodes and blood, including development of stem-like progenitor Tfh cells. However, aging induced rewiring in the later stages of Tfh development, culminating in transcriptionally distinct effector Tfh cells with features of augmented effector states, cytokine production, and cellular senescence. Extrinsically, the inflammaging microenvironment was sufficient to induce some of these changes in late effector Tfh differentiation, including the inability to transition to a resting state, pro-inflammatory cytokine production, and a cellular senescence-like state. These studies demonstrate that humoral immunity is uniquely wired in aging rather than simply an attenuated version of a youthful immune system.

## Results

### Tfh cells clonally expand in lymph nodes and blood after influenza vaccination.

**Figure 1:**
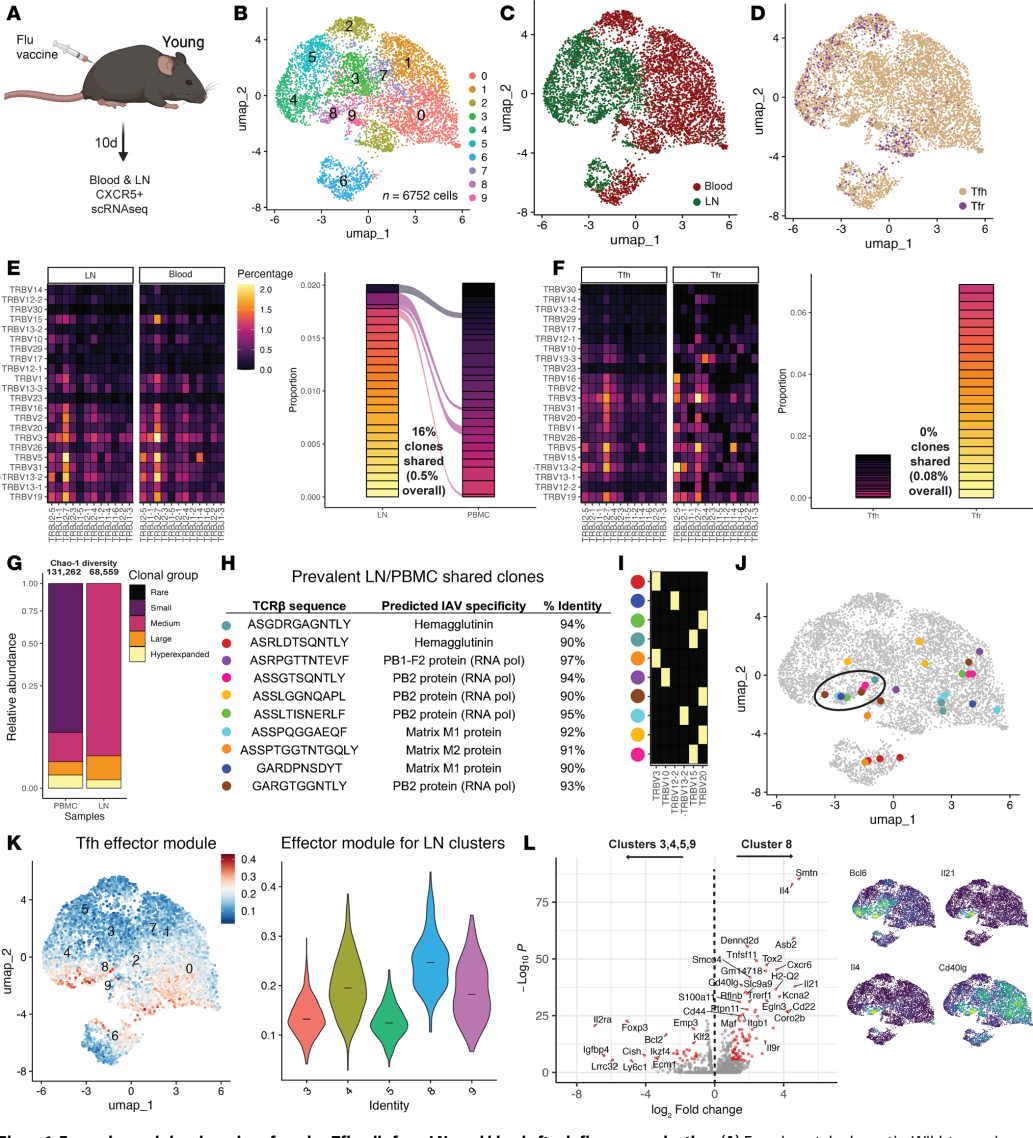
Expansion and clonal overlap of murine Tfh cells from LNs and blood after influenza vaccination. (**A**) Experimental schematic. Wild-type mice (8–12 weeks of age) were administered the Afluria quadrivalent influenza vaccine (total 1.5 μg HA protein). After 10 days, CD4^+^CXCR5^+^ cells were sorted from both dLN and peripheral blood, and single-cell RNA sequencing (scRNA-Seq) with matched TCR-Seq was performed. (**B**–**D**) UMAPs of *n* = 6,752 cells by unsupervised cluster assignment (**B**), by tissue (**C**), and by cell type (**D**). (**E**) (Left) TCRβ-V and J gene usage. Percentage indicates frequency of use in dataset. (Right) Clonal sharing between the top 25 TCR clones by prevalence in LN and blood. Connecting lines indicate shared clones. (**F**) (Left) TCRβ V-J gene segment usage and (right) clonal sharing between Tfh and Tfr cells. (**G**) Clonal expansion (<0.01% “rare,” 0.01%–0.033% “small,” 0.033%–0.067% “medium,” 0.067%–0.1% “large,” and >0.1% “hyperexpanded”) for LN and blood cells. (**H**) TCR complementarity-determining region 3 amino acid sequence and predicted influenza specificity of the top 10 clones shared between LN and blood Tfh cells. Each clone is color coded. (**I**) TCRβ -V gene usage for clones in **H**. (**J**) UMAP of clones from **H**, including annotation of cluster 8 from **B**. (**K**) Module score for a Tfh Effector module (derived from ref. 14) expressed as a feature plot (left) or violin plot (right). Violin plot displays the Tfh Effector feature score 95% confidence interval for cells in indicated clusters, with the shape indicating the probability density and horizontal line denoting the median value. (**L**) (Left) Differential gene expression between cluster 8 and clusters 3, 4, 5, and 9 of the LN. (Right) Density plots for indicated genes. Data are from a single scRNA-Seq experiment of 2 individual mice concatenated.

Differentiation of Tfh cells has recently been shown to require sequential developmental stages in lymphoid organs, such as the draining lymph node (dLN), after vaccination (14). Some Tfh cells can leave the dLN and enter the circulation before full differentiation, but the relationship between these populations and those that reside in LNs is not fully understood. Thus, we first assessed the clonal relationships of Tfh cells in dLNs and blood of influenza-vaccinated mice. We administered an unadjuvanted quadrivalent influenza vaccine (Afluria Influenza A+B, 2010–2011 formulation) to 8-week-old C57BL/6 mice and 10 days later sorted total follicular T cells (gated as CD4^+^CXCR5^+^CD19^–^; Tfh and Tfr together) from the dLN and blood (Supplemental Figure 1A; supplemental material available online with this article; https://doi.org/10.1172/jci.insight.187271DS1). Cells were processed for single-cell RNA-Seq (scRNA-Seq) with matched TCR-Seq (Figure 1A). The 6,752 follicular T cells that passed quality control filters separated into 10 distinct clusters in uniform manifold approximation and projection (UMAP) space (Figure 1B). Tissue origin was a dominant factor for the positioning of cells in clusters, since follicular T cells from the dLN separated from the blood (Figure 1C). Only a single cluster, cluster 6, contained cells from both the dLN and blood. However, even within this cluster, we observed evidence of segregation. We then separated the follicular T cells into Tfh and Tfr cells by assigning cells that expressed *Foxp3* or a T regulatory cell–dominant (Treg-dominant) transcriptional profile as Tfr cells (Supplemental Figure 1, B and C). Tfr cells were found in cluster 2 of the blood, as well as clusters 4 and 5 of the dLN, whereas Tfh cells were found in all clusters (Figure 1D). Notably, the tissue of origin was a stronger overall driver of transcriptional programs than Tfh versus Tfr cell type, consistent with previous findings (25).

Reclustering of the 5,352 Tfh and 1,391 Tfr cells independently revealed separation of dLN and blood populations for both cell types (Supplemental Figure 1, E–K). Analysis of differentially expressed genes (DEGs) between dLN and blood Tfh/Tfr cells identified 103 tissue-specific genes in common between Tfh and Tfr, suggesting similar tissue-specific transcriptional programming (Supplemental Figure 1K). Genes enriched in the blood compartment included some consistent with a quiescent state (*Klf3*, *Dusp1*). In contrast, genes similarly enriched in dLN included genes associated with effector Tfh cells (*Bcl6*, *Pdcd1*/PD-1, *Id3*), as well as costimulatory molecules (*Tnfrsf4*/OX40, *Tnfrsf9*/4-1BB).

Next, we compared TCR sequences to determine clonal overlap of Tfh cells in the dLN and blood after influenza vaccination. Overall, TCRβ chain V-J usage was similar in follicular T cell clones between dLN and blood, suggesting clonal overlap (Figure 1E). Moreover, 0.5% of all, and 16% of the most abundant, clones were shared with expanded Tfh clones in the blood, further supporting clonal overlap of Tfh cells after influenza vaccination (Figure 1E). Although the vast majority of Tfr cells are thought to originate from natural Tregs, some studies have suggested a small portion of Tfr cells can originate from non-Tregs as “induced” Tfr cells (26). However, consistent with prior reports (27), we found that TCRβ gene usage was distinct between Tfh and Tfr cells. Furthermore, we observed minimal overlap of Tfh clones with dLN or blood Tfr cells, with only 0.08% of Tfr clones overlapping with Tfh cells (Figure 1F). Notably, all Tfr clones were singletons, and there was no evidence of clonal proliferation (Figure 1F).

The lower amount of clonal expansion in blood, compared with dLN, Tfh cells suggests circulating cells have more clonal diversity (Figure 1G). Despite differences in the extent of clonal expansion, a small subset of Tfh clones were expanded in both the dLN and blood. Of the top 10 most expanded clones in both tissues, all were predicted to have reactivity for influenza A virus. The most common predicted influenza epitopes included HA, RNA polymerase, and matrix proteins (Figure 1H), though the top predicted specificity for a few expanded clones was for other viruses or autoantigens (Supplemental Figure 1L). Expanded Tfh clones made use of a diversity of TCRβ V-gene segments; however, V20 was the most common (Figure 1I). These Tfh clones were found in clusters 8 and 9 of the dLN as well as most Tfh clusters of the blood (Figure 1J). Cluster 8 had enrichment of a Tfh effector gene module (derived from TfhFull vs. Tfh progenitor-like, or TfhProg, cells) (14), suggesting this population is a fully differentiated effector Tfh population (Figure 1K). Consistent with this, Tfh effector genes *Il21*, *Maf*, *Bcl6*, *Il4*, and *Cd40lg* were among the most differentially expressed genes in this cluster (Figure 1L) (14). Together, these data indicate that Tfh cells undergo sequential development in the dLN after influenza vaccination, culminating in an effector Tfh state, and that some of these expanded clones gain access to the blood to recirculate.

### Tfh cells from aged mice undergo transcriptional rewiring during later stages of development to induce a cellular senescence-like program.

**Figure 2:**
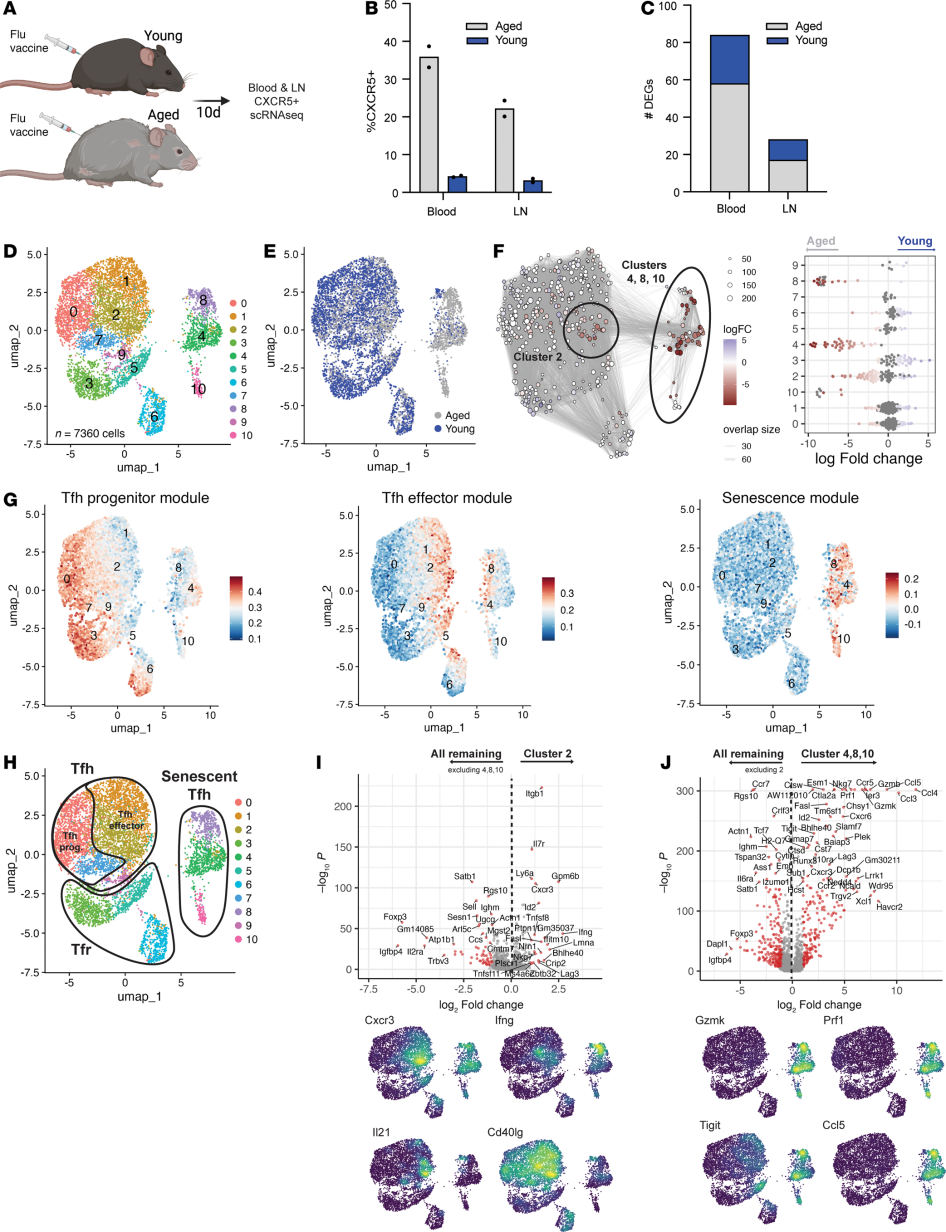
Tfh cells from aged mice have rewired progressive differentiation culminating in a cellular senescence-like program. (**A**) Experimental schematic. Aged (80 weeks) mice underwent quadrivalent influenza vaccination as in Figure 1 and were processed for scRNA-Seq/TCR-Seq, and data were added to data from young mice (Figure 1). (**B**) Quantification of total CD4^+^CXCR5^+^ follicular T cells. (**C**) Number of differentially expressed genes (DEGs) between young and aged total follicular T cells, separated by tissue. Color indicates directionality of fold-change. (**D** and **E**) UMAP of blood follicular T cells by unsupervised clustering (**D**) and annotated for group (**E**). (**F**) miloR cell neighborhood analysis illustrating differential abundance of cell neighborhoods in young (blue) versus aged (red) mice, represented in UMAP space or by individual cluster (FDR < 0.05, fold-change > 2). (**G**) Module score feature plots for a Tfh Progenitor module (derived from ref. 14), Tfh Effector module (derived from ref. 14), or senescence module (29). (**H**) Annotation of clusters in UMAP space based on cell type and/or phenotype. (**I**) (Top) DEGs in cluster 2 versus clusters 0, 1, 3, 5–7, 9. Red dots indicate *P* < 10^–2^, fold-change > 2. (Bottom) Density plots for indicated DEGs. (**J**) (Top) DEGs between clusters 4, 8, and 10 versus clusters 0, 1, 3, 5–7, 9. Red dots indicate *P* < 10^–2^, fold-change > 2. (Bottom) Density plots for selected DEGs. Data are concatenated data from 2 individual mice in each age group.

Aging can affect the quality of the antibody response elicited by influenza vaccination (1–5). Passive infusion of serum from young, influenza-vaccinated mice was protective against severe influenza infection, whereas infusion of serum from aged, vaccinated mice was not (Supplemental Figure 2). To investigate the mechanism of this observed deficiency, we vaccinated both young and 80-week-old (aged) mice to assess how settings of aging alter progressive Tfh differentiation after influenza vaccination utilizing scRNA-Seq with matched TCR-Seq (Figure 2A). Consistent with previous reports (19, 28), the frequency of total CD4^+^CXCR5^+^ follicular T cells as determined by flow cytometry was higher in aged versus young vaccinated mice (Figure 2B). The number of DEGs between young and aged follicular T cells was calculated separately for each tissue. We found a greater number of DEGs (corrected *P* < 0.01; fold-change > 2) in the blood compared with the dLN (Figure 2C). We focused further analysis on blood Tfh cells, since 1) we found stronger age-related transcriptional changes in blood Tfh cells, 2) we found more diverse phenotypes of expanded Tfh cells in the blood of young mice, and 3) most human Tfh studies have access to blood only for analysis. We combined 7,360 CD4^+^CXCR5^+^ follicular T cells from the blood of young or aged mice. These cells segregated into 11 unique clusters in UMAP space (Figure 2D and Supplemental Table 1). There were clear differences in the distribution of young and aged cells in clusters, with miloR neighborhood analysis revealing that follicular T cells from aged mice disproportionately occupied clusters 2, 4, 8, and 10 (Figure 2, E and F). In contrast, cluster 3, and to a lesser extent cluster 6, were disproportionately occupied by young follicular T cells. Reclustering of 6,600 Tfh and 755 Tfr cells separately demonstrated that the differences between young and aged follicular T cells was due to Tfh cells (Supplemental Figure 3).

Next, we assessed whether changes in Tfh cells in aged mice occur during early or later stages of Tfh differentiation. To do this, we utilized gene modules for progenitor-like and fully differentiated effector Tfh cell states (14). Youth-associated clusters 3 and 6 had enrichment of a progenitor-like Tfh module, as did clusters 0 and 7, which were not altered between young versus aged mice (Figure 2G). In contrast, age-associated clusters 2, 8, and 4 had enrichment of a fully developed effector Tfh gene module, as did clusters 1 and 5, which were not altered with age (Figure 2G). Age-related transcriptional changes in nonfollicular T cell subsets have been linked to cellular senescence (29). To determine if transcriptional rewiring in aged Tfh cells included a cellular senescence program, we assessed a gene module (incorporating conserved tissue signatures) for this program (29) and found strong enrichment in age-associated clusters 4, 8, and 10 (Figure 2, G and H). Several individual “senescence” genes from the gene module were increased in aged cells (e.g., *Ccl4*, *Ccl3*, *Icam1*, *Tnf*), and a few showed minor increases in young cells (*Pecam1*, *Cd55*, *Il6st*, *Jun*) (Supplemental Table 2). Together, these data suggest that aged mice have disproportionately more effector-like Tfh cells that are rewired.

To understand mechanisms contributing to age-related transcriptional rewiring of Tfh differentiation, we assessed genes overabundant in cluster 2, a cluster that was enriched in aging but also populated by Tfh cells from young mice (Figure 2I). This cluster had enrichment for effector Tfh genes and cytokines such as *Cxcr3*, *Ifng*, *Il21*, and *Cd40lg* (Figure 2I). In contrast, clusters 4, 8, and 10, which were age- and senescence-associated, were enriched for cytotoxic genes (*Gzmk*, *Prf1*/Perforin), coinhibitory receptors (*Pdcd1*/PD-1, *Lag3*, *Tigit*, and *Havcr2*/Tim-3), and individual senescence genes (*Ccl3*, *Ccl4*, *Ccl5*) (Figure 2J). Moreover, clusters 4, 8, and 10 had lower expression of negative regulators of Tfh cells, such as *Satb1*, *Klf2*, *Foxo1*, and the progenitor-insulating transcription factor *Foxp1*. Similar results were found in the LN Tfh compartment, indicating similar rewiring in both LN and blood (Supplemental Figure 4).

### Clonal origins of age-rewired Tfh cells.

**Figure 3:**
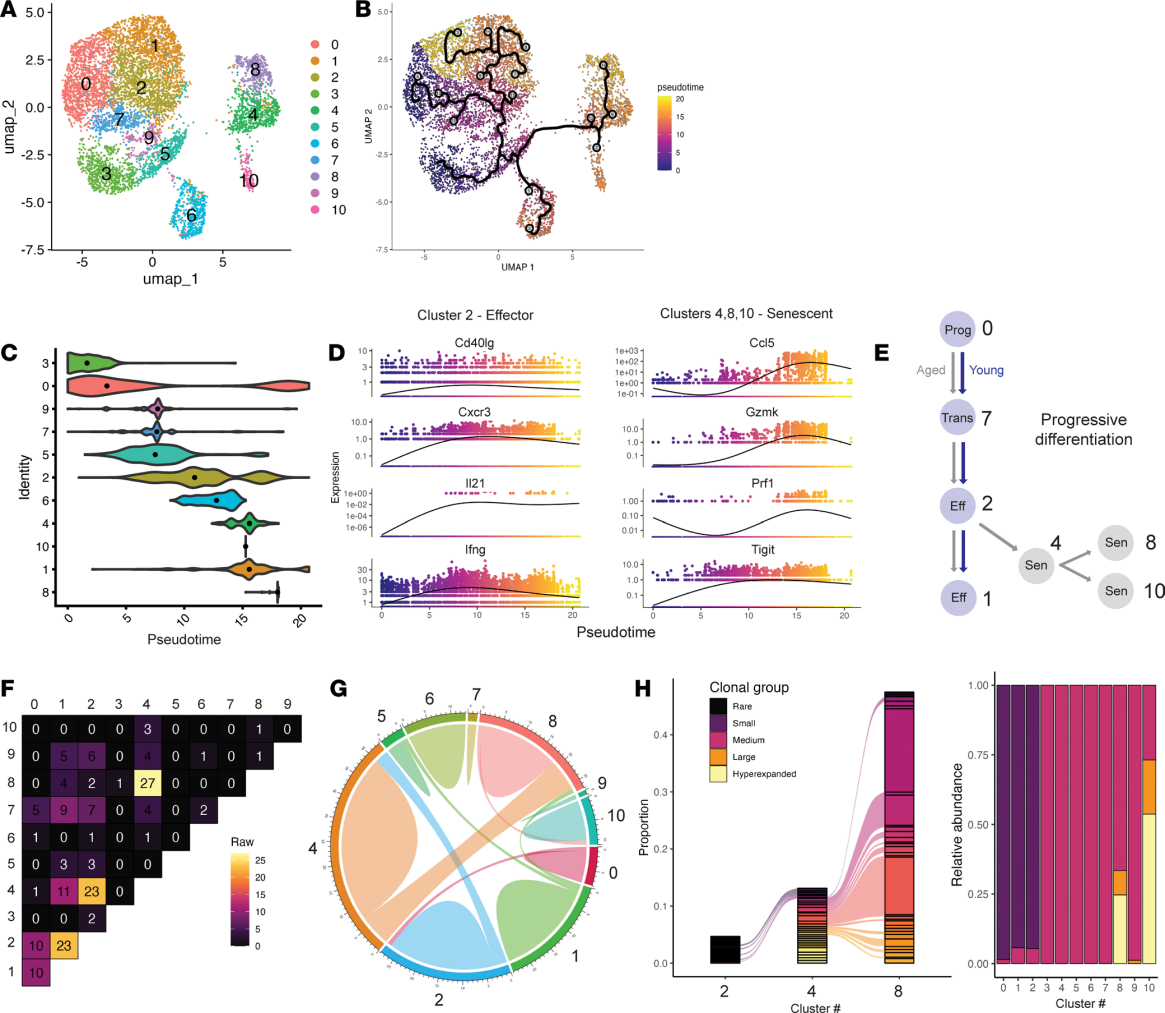
Clonal origins of age-rewired Tfh cells in mice. (**A** and **B**) UMAP of *n* = 7,360 blood follicular T cells by unsupervised clustering (**A**) and Monocle3 pseudotime trajectories (**B**), with root nodes set for progenitor clusters 0 and 3. Black ribbon indicates projected path of differentiation from progenitor population. (**C**) Pseudotime values per cluster with black dot representing median. (**D**) Gene expression values of indicated genes organized by ascending pseudotime values. (**E**) Schematic of progressive differentiation in Tfh cells from young or aged mice with cluster annotation. (**F**) Clonal sharing (based on TCR sequence) including absolute number of shared clones between clusters (left). (**G**) Circos plot of clonal sharing of expanded Tfh clones (shared by >2 individual cells). (**H**) (Left) Top 25 expanded clones in individual clusters 2, 4, and 8, with connecting lines indicating shared clones. (Right) Clonal expansion per cluster (<0.01% “rare,” 0.01%–0.1% “small,” 0.1%–1% “medium,” 1%–5% “large,” and >5% “hyperexpanded”).

We next determined the clonal origins of age-rewired senescent-like Tfh cells. Monocle3 pseudotime analysis was performed on the scRNA-Seq datasets using the progenitor Tfh cluster 0 as the root node. We found evidence of progressive development from the progenitor Tfh population through cluster 7 and subsequently to cluster 2 (Figure 3, A–C). Interestingly, cells within cluster 2 showed developmental branching/fate decisions with bifurcation to either cluster 1 (an effector Tfh cluster) or toward age-associated clusters 4, 8, and 10. Notably, these age-associated clusters had the highest pseudotime values, indicating the most terminal differentiation. Analysis of young and aged Tfh cells independently verified that senescence-like clusters had the highest pseudotime values (Supplemental Figure 5). We also found evidence of sequential differentiation from effector-like cluster 1 toward progenitor-like cluster 0, possibly indicating the presence of TfhEx cells that dampen the effector program but remain epigenetically poised (14). At the individual-gene level, the expression of cluster 2–associated genes, including *Il21*, *Cxcr3*, *Cd40lg*, and *Ifng*, increased along pseudotime trajectories, eventually reaching a plateau at an intermediate pseudotime (Figure 3D). In contrast, expression of genes associated with clusters 4, 8, and 10, such as *Ccl5*, *Gzmk*, and *Prf1*, abruptly increased only during later pseudotime values (Figure 3D). However, the exception was *Tigit*, which gradually increased similar to the behavior of cluster 2 genes. These data suggest that aged effector Tfh cells are rewired from normal effector differentiation toward a senescence-like transcriptional state (Figure 3E).

To further interrogate the developmental relationships between age-associated senescent clusters 4, 8, and 10 with other Tfh cells, we analyzed TCR sequences. Cluster 2, which represented a branch point in Tfh differentiation, had clonal overlap with both Tfh Effector cluster 1 as well as age-senescent cluster 4, further indicating important fate decisions during this stage (Figure 3, F–H). We also found clonal sharing between clusters 1 and 4, though to a lesser degree, indicating that the same Tfh clones could undergo further differentiation from cluster 2 to *either* effector Tfh or age-rewired Tfh cells. We also found robust clonal sharing between the 3 age-rewired/senescent-like clusters 4, 8, and 10. Interestingly, even though we found substantial clonal overlap between clusters 2 and 4, as well as clusters 8 and 4, we found minimal clonal sharing between clusters 2 and 8. These data indicate that age-rewired Tfh cells may arise by redirecting effector stages of Tfh differentiation (e.g., through cluster 2) or possibly by age-rewired non-Tfh populations differentiating into Tfh cells (e.g., cluster 8). Intriguingly, age-rewired clusters 8 and 10 exhibited substantial clonal expansion and were dominated by hyperexpanded clones, some of which had predicted specificity for murine cytomegalovirus protein epitopes (data not shown). Notably, the senescent-like Tfh cell population still expressed canonical Tfh cell markers, including *Cxcr5*, *Bcl6*, *Pdcd1*, *Icos*, and *Il21* (Supplemental Figure 6). Last, we found a minor degree of clonal sharing between Tfh cluster 1 and Tfr cluster 5. These cells could represent ex-Tfr cells that have downregulated their Tfr transcriptional program or alternatively induced Tfr cells from Foxp3-negative precursors (25). Together, these data indicate that in murine models of aging, Tfh cells undergo normal early differentiation, but later stages of Tfh differentiation are redirected toward an age-rewired state with evidence of hyperactivation, cytokine production, and senescence-like programs.

### Defective neutralizing antibody and ICOS^hi^CD38^hi^ Tfh cells in older humans after influenza vaccination.

**Figure 4:**
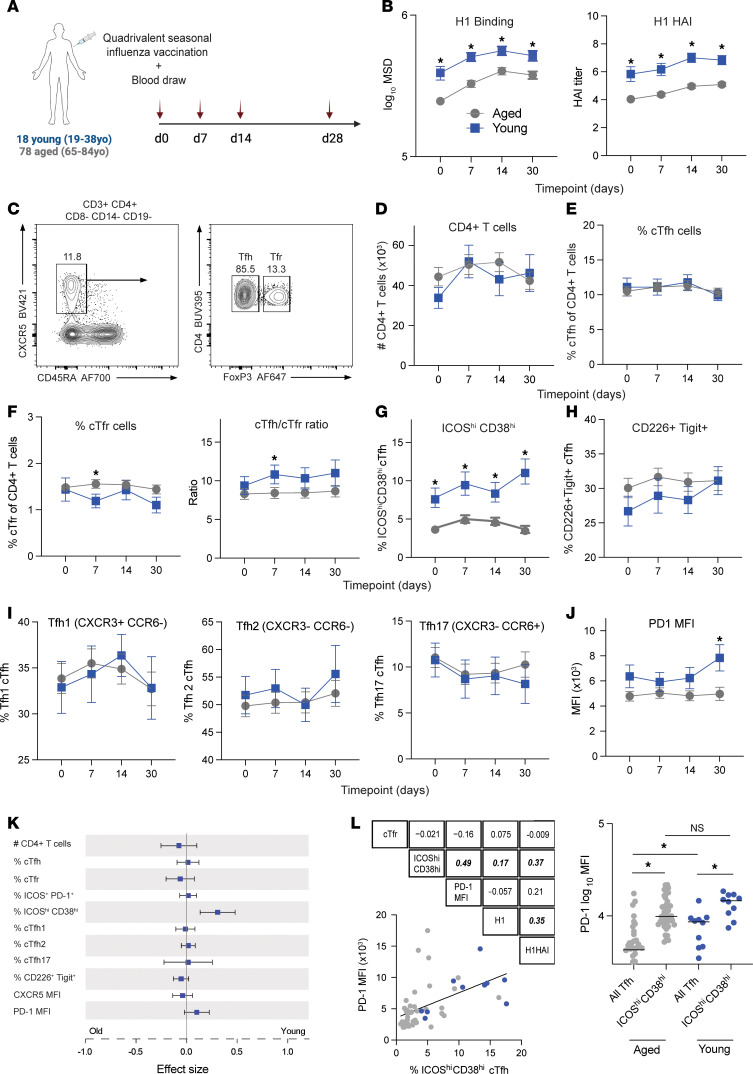
Defective neutralizing antibody and ICOS^hi^CD38^hi^ Tfh cells in older humans after influenza vaccination. (**A**) Experimental schematic. 18 young (18–38 yr) and 78 aged (> 65 yr) adults were administered a seasonal influenza vaccine. Peripheral blood was obtained on the day of vaccination (d0), as well as day 7 (d7), day 14 (d14), and day 30 (d30) after vaccination. (**B**) (Left) Influenza H1 protein Meso Scale Discovery (MSD) binding index for young and aged vaccine recipients, separated by time point. (Right) Influenza H1 protein hemagglutination inhibition (HAI) titers. (**C**) Gating strategy to identify cTfh (CD3^+^CD4^+^CD8^–^CD14^–^CD19^–^CD45RA^–^CXCR5^+^FoxP3^–^) and Tfr (CD3^+^CD4^+^CD8^–^CD14^–^CD19^–^CD45RA^–^CXCR5^+^FoxP3^+^) cells from blood. (**D**) Total number of CD4^+^ T cells (per 1 × 10^6^ input PBMC), separated by time point. (**E**) Tfh frequency of total CD4^+^ T cells, separated by time point. (**F**) Tfr frequency of total CD4^+^ T cells (left) and Tfh/Tfr ratio (right), separated by time point. (**G** and **H**) ICOS^hi^CD38^hi^ (**G**) or CD226^+^Tigit^+^ (**H**) frequency of Tfh cells, separated by time point. (**I**) Tfh cell polarization including Tfh1 (left), Tfh2 (middle), and Tfh17 (right) identified by indicated chemokine receptor expression. (**J**) PD-1 mean fluorescence intensity on total Tfh cells, separated by time point. (**K**) Forest plot indicating longitudinal modeling of indicated parameters (#CD4^+^ T cells, %cTfh, %cTfr, %ICOS^+^PD-1^+^, %ICOS^hi^PD-1^hi^, %cTfh1, %cTfh2, %cTfh17, %CD226^+^Tigit^+^, CXCR5 MFI, and PD-1 MFI). (**L**) Spearman’s rank correlation for indicated parameters of interest. Linear regression of %ICOS^hi^CD38^hi^ cells with PD-1 MFI, for all cells at d30 time point. Bold italic indicates statistical significance. (Right) PD-1 MFI on total or ICOS^hi^CD38^hi^ Tfh cells. All displayed data points represent mean, with error bars indicating SEM. *P < 0.05, uncorrected Mann-Whitney *U* test.

To assess how aging alters Tfh differentiation and function in humans, we obtained peripheral blood from 18 young (18–38 years) and 78 aged (>65 years) individuals on days 0, 7, 14, and 30 after seasonal influenza vaccination (Figure 4A and Supplemental Table 3). The distribution of men and women was similar between young (56% female) and aged (69% female) cohorts (*P* = 0.24, χ^2^ test). Baseline binding of serum IgG to the influenza vaccine H1 protein was higher in young versus aged individuals, even at baseline (mean log_10_ Meso Scale Discovery [MSD] binding index in aged = 4.29, young = 4.54, *P* = 0.003, Mann-Whitney *U* test) (Figure 4B). Analogously, the vaccine H1 serotype HA inhibition (HAI) titer was higher in young versus aged individuals at baseline as well as at day 14 after vaccination, which was the peak of response. Though aged participants may have received either the standard or high-dose vaccine, notably, there was no dose-dependent difference in MSD titer observed in this cohort (mean log_10_ MSD for aged participants receiving standard dose = 4.49 vs. high dose = 4.48). Notably, there was no difference in H1 binding or HAI between male and female participants, even when separated by age group (Supplemental Table 4).

Next, we utilized high-parameter spectral flow cytometry to investigate whether total or subsets of Tfh cells were altered in aged individuals. The total number of CD4^+^ T cells was indistinguishable between young and aged individuals (Figure 4, C and D). Circulating follicular T cells (defined as CD3^+^CD4^+^CD8^–^CD14^–^CD19^–^CD45RA^–^CXCR5^+^) were separated based on FoxP3 expression into circulating Tfh (FoxP3^–^) and circulating Tfr (cTfr) (FoxP3^+^) cells (Figure 4C and Supplemental Figure 7). The frequency of total cTfh cells was not significantly different between young and aged individuals at any time point (Figure 4E). cTfr cells were similar at baseline between young and aged individuals but exhibited a transient decrease at day 7 after vaccination in young individuals only (mean frequency aged = 1.56%, young 1.19%, *P* = 0.046, Mann-Whitney *U* test) (Figure 4F). The ratio of Tfh/Tfr, which predicts vaccine responses in some settings (30), was slightly higher in young versus aged individuals starting at day 7 after vaccination (d7 mean ratio aged = 8.42, young = 10.82, *P* = 0.029, Mann-Whitney *U* test) (Figure 4F).

Since we did not find changes in total cTfh cells, we next determined whether developmental stage, as indicated by activation state, was different between young and aged individuals. We found higher proportions of ICOS^hi^CD38^hi^ Tfh cells (previously shown to correlate with influenza vaccination) (31, 32) in young versus aged individuals at all time points (Figure 4G). In contrast, we did not find any statistical differences in CD226^+^Tigit^+^ Tfh or Tfh1-like (CXCR3^+^CCR6^–^) cells, which have been associated with influenza vaccine responses (Figure 4, H and I) (33, 34). Moreover, we did not find any differences in other cytokine-polarized Tfh cells, such as Tfh2-like cells (CXCR3^–^CCR6^–^) or Tfh17-like cells (CXCR3^–^CCR6^+^) (Figure 4I) (35). In addition, we assessed levels of programmed cell death 1 (PD-1) on Tfh cells, since PD-1 is a negative regulator of Tfh cell differentiation and function but is highly expressed in later effector stages (36, 37). We found higher expression of PD-1 in young individuals; however, this only reached statistical significance 30 days after influenza vaccination (Figure 4J). There was no statistical difference in the change of each of these parameters from baseline values (Supplemental Figure 8). Longitudinal data for each of these parameters were modeled using nonlinear mixed methods to assess changes between groups. This analysis revealed increases in the ICOS^hi^CD38^hi^ Tfh population in young versus aged influenza vaccinees (Figure 4K). Spearman’s rank analysis indicated that the ICOS^hi^CD38^hi^ Tfh population correlated with serological H1 binding, serological H1 HAI, and Tfh PD-1 expression (Figure 4L). Together, these data indicate that the activation state, and not total frequency, of Tfh cells may be altered in an age-dependent manner in humans after influenza vaccination, implicating changes in sequential differentiation.

### Human Tfh cells undergo progressive differentiation after influenza vaccination.

**Figure 5:**
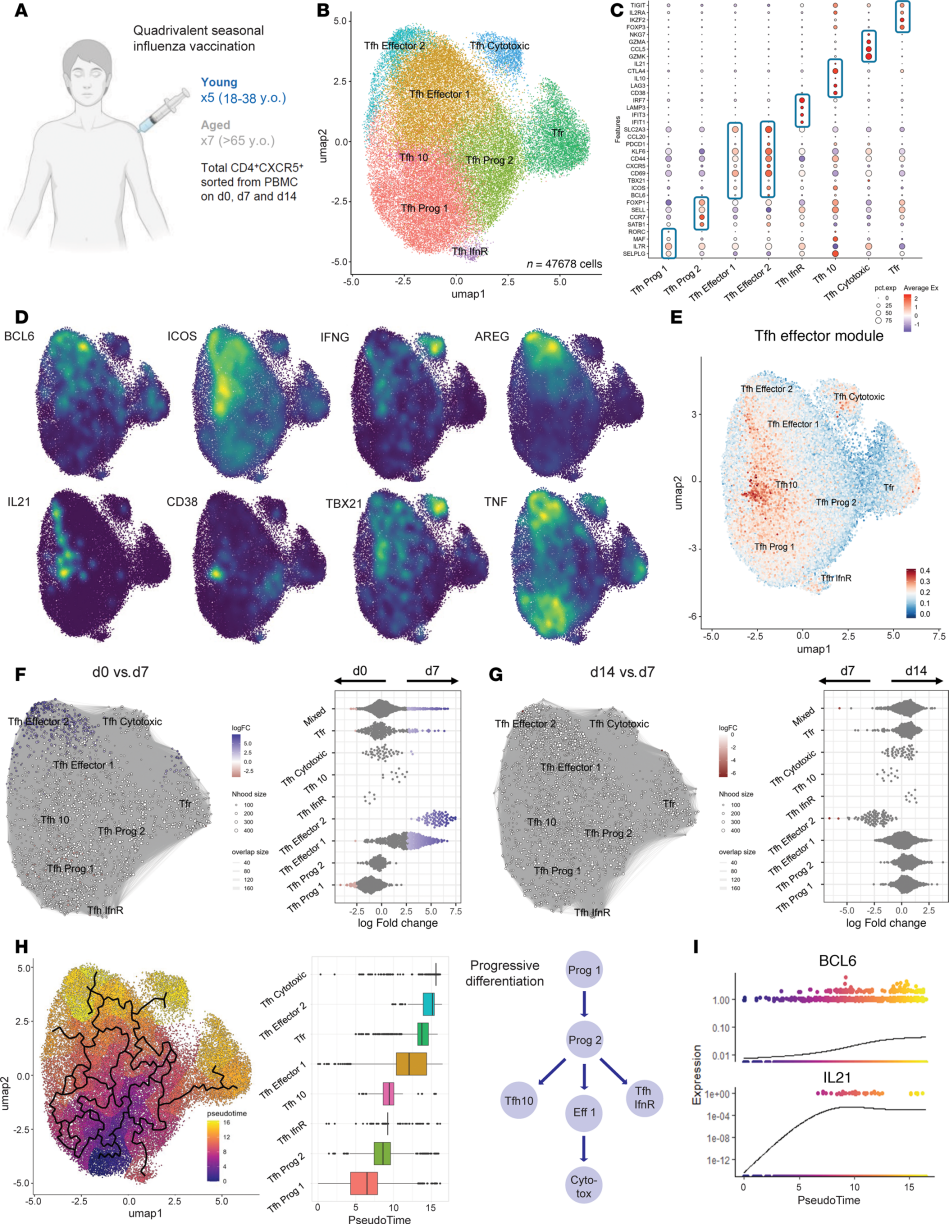
Human Tfh cells undergo progressive differentiation after influenza vaccination. (**A**) Experimental schematic. Total CD4^+^CXCR5^+^ T cells were sorted from blood of young or aged individuals on day 0, 7, or 14 after seasonal influenza vaccination. (**B** and **C**) UMAP of *n* = 47,678 cells integrated across patients and time points, with clusters annotated based on genes involved in sequential Tfh development. (**D**) UMAP showing transcript density for indicated genes. (**E**) Module score feature plot for a Tfh Effector module (14). (**F** and **G**) miloR differential neighborhood abundance assessment of total CD4^+^CXCR5^+^ T cells from all donors at indicated days. Node size indicates the number of cells in the neighborhood; color denotes direction of differential abundance (miloR, FDR 0.10). (**H**) (Left) UMAP and cluster box plot of Monocle3 pseudotime analysis utilizing a root node in the Tfh Prog 1 cluster. Color brightness denotes the predicted lapsed pseudotime for cluster differentiation. Black ribbon approximates the anticipated differentiation pathway. (Middle) Pseudotime values per cluster, with black bar representing median. (Right) Proposed progressive differentiation pathway for Tfh. (**I**) Expression values of *BCL6* and *IL21* ordered by pseudotime values. Data shown in **F** and **G**, right, are expressed as single data points; data shown in **H**, right, are expressed as box and whiskers (median, IQR, and range).

Since our flow cytometric data indicated age-related changes in Tfh activation/developmental states more so than total Tfh cell frequencies, we sought to understand Tfh differentiation in humans after influenza vaccination. We performed scRNA-Seq on Tfh and Tfr (by sorting total CD4^+^CD19^–^CXCR5^+^) cells from peripheral blood of 5 young and 7 aged individuals at baseline, day 7, and day 14 after seasonal influenza vaccination (Figure 5A and Supplemental Figure 9A). A total of 47,678 cells passed quality control filters and subsequently formed 8 distinct clusters in UMAP space (Figure 5B). Most clusters lacked expression of *FOXP3* and were designated as Tfh clusters, whereas a single cluster showing high expression of *FOXP3* and *IKZF2* was annotated as Tfr. Based on genes associated with Tfh developmental stages (14), we identified 2 progenitor-like Tfh clusters (Tfh Prog 1 and Tfh Prog 2) marked by expression of *SELPLG*, *MAF*, *CCR7*, *SELL*, *IL7R*, or *FOXP1*, along with 2 effector-like Tfh clusters (Tfh Effector 1 and Tfh Effector 2) in which *ICOS*, *CD44*, *CXCR5*, and *CD69* were upregulated (Figure 5, B and C). We also observed a small cluster marked by expression of *IL10*, *CTLA4*, and *IL21*, referred to as Tfh10 consistent with previous reports (38). In addition, we identified Tfh clusters marked by expression of interferon-responsive elements (*IFIT1*, *IFIT3*, *IRF7*) referred to as Tfh IfnR, as well as a cytotoxic cluster (*GZMA*, *GZMK*, *CCL5*, *NKG7*, *IFNG*) referred to as Tfh Cytotoxic, both of which have been previously reported (Figure 5C) (39–41). Notably, the ICOS^hi^CD38^hi^ population of Tfh identified via flow cytometry likely correspond to Tfh Effector 1 and Tfh10 clusters (Figure 5D), which is consistent with a Tfh effector-like transcriptional program (Figure 5E). Furthermore, *IFNG* and *TBX21*, which encodes the transcription factor T-bet, were present in both Tfh Effector 1 and Effector 2 clusters, as well as the Tfh Cytotoxic cluster (Figure 5D). Last, the expression of several senescence-associated genes, such as *AREG* and *TNF* (29), was enhanced in the Tfh Effector 2 cluster.

Differential abundance testing across cell neighborhoods revealed that the Tfh Effector 1 and 2 clusters were more abundant on day 7 after vaccination compared with prevaccine levels (k = 45, d = 20, FDR = 0.10), suggesting the Tfh Effector programs were induced by vaccination (Figure 5F). Moreover, Tfh Prog 1 was more abundant at baseline compared with day 7. In contrast, when we assessed relative abundance between days 7 and 14 after vaccination, we found only a minor reduction in the Tfh Effector 2 cluster at day 14, indicating that most Tfh cells maintained their programs until at least day 14 (Figure 5G). We next conducted pseudotime analyses to assess differentiation relationships between clusters. We found Tfh Prog 1 and 2 clusters had the lowest pseudotime values consistent with their progenitor designation (Figure 5H). Tfh IfnR and Tfh10 clusters had intermediate pseudotime values, and Tfh Effector 1 and 2, Tfh Cytotoxic, and Tfr clusters had the highest pseudotime values (Figure 5H). These data indicate progressive development of Tfh cells, culminating in effector-like Tfh cells in the circulation (Figure 5H). The expression of *IL21* and *BCL6* along the pseudotime trajectory was also consistent with progressive differentiation (Figure 5I). Next, we compared genes differentially expressed between the most progenitor-like Tfh cluster, Tfh Prog 1, and the 2 effector Tfh clusters. Effector Tfh clusters had higher expression of essential Tfh genes, such as *BCL6* and *ICOS*, compared with progenitor Tfh cells, as well as reduced expression of the progenitor-strengthening transcription factor *FOXP1* (Supplemental Figure 9B). Together, these data indicate Tfh cells undergo progressive development in humans after influenza vaccination.

### Tfh cells from older individuals undergo transcriptional rewiring during late effector stages.

**Figure 6:**
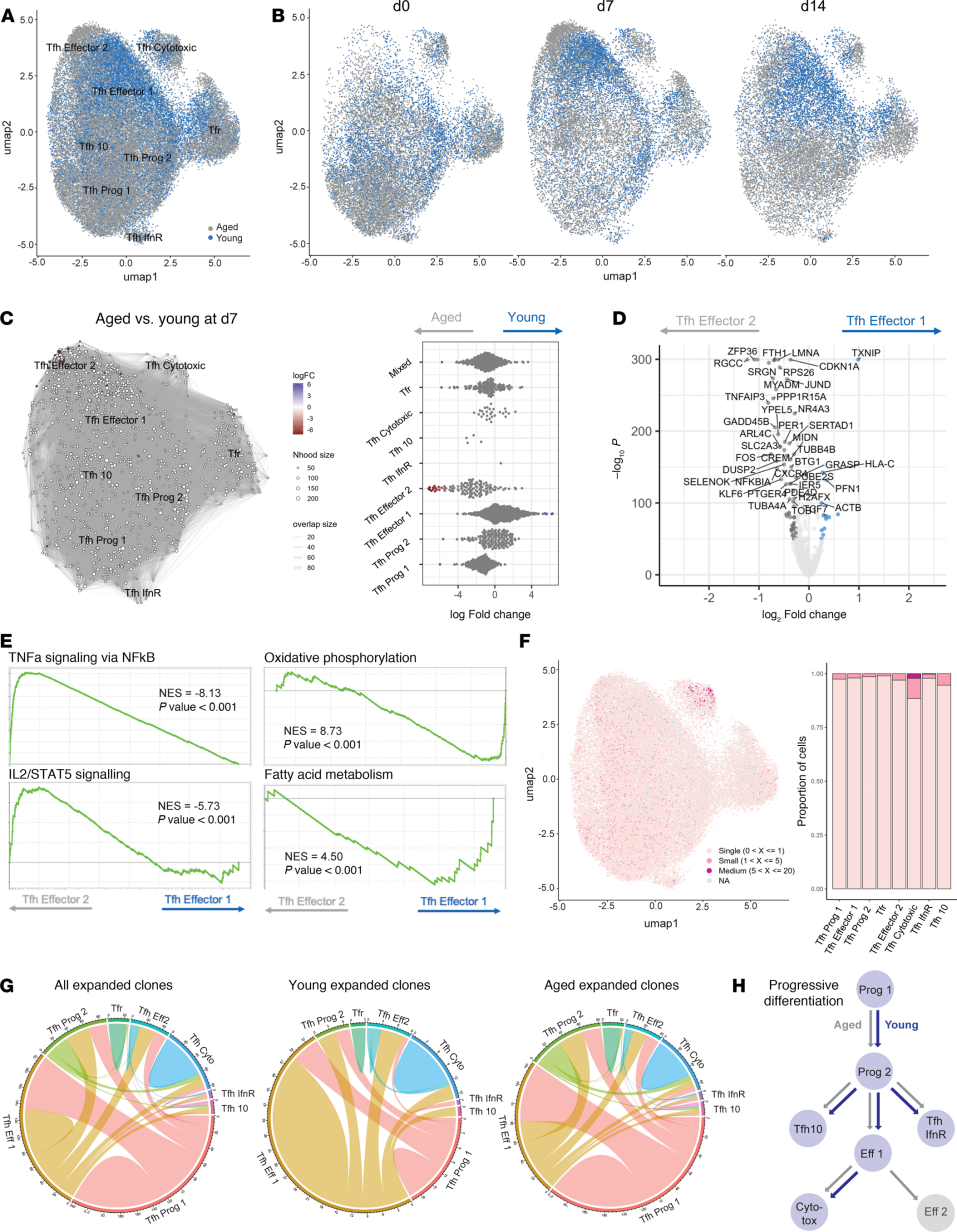
Tfh cells from older individuals undergo transcriptional rewiring during late effector stages. (**A**) UMAP of total human Tfh cells from Figure 5A separated according to age (blue: young; gray: aged) and (**B**) split according to time point after vaccination. (**C**) miloR neighborhood analysis representing differential abundance between Tfh cells from aged and young individuals 7 days after vaccination (miloR, FDR 0.25). Node size indicates the number of cells in the neighborhood; color denotes direction of differential abundance. (**D**) DEGs between Tfh Effector 1 and Tfh Effector 2 clusters (cutoffs: fold-change = 0.25, *P* = 10^–50^; Wilcoxon’s rank sum test, presto package). (**E**) GSEA enrichment of selected Hallmark gene modules in Tfh Effector 1 versus Tfh Effector 2 (NES, normalized enrichment score). (**F**) UMAP and summary of clonal expansion in Tfh cells across clusters. (**G**) Circos plots representing clonal sharing of expanded clones among clusters for all participants (left), young participants (middle), and aged participants (right). (**H**) Proposed progressive differentiation pathway for young and aged Tfh.

To assess possible alterations in Tfh differentiation in aged individuals after influenza vaccination, we separated cells into young (18–65 years) or aged (>65 years) origins (Figure 6, A and B). In total, our data set included 31,009 cells from 7 aged donors and 16,669 cells from 5 young donors. Young and aged Tfh cells segregated in Tfh Effector clusters. Differential abundance testing based on cell neighborhoods at day 7 after vaccination identified enrichment of Tfh Effector 1 cells in young individuals and enrichment of Tfh Effector 2 cells in aged individuals (k = 25, d = 20, FDR = 0.25) (Figure 6C). No substantial changes in abundance were identified prevaccination (data not shown) or in Tfh progenitor populations at day 7 after vaccination (Figure 6C), suggesting age-related changes in Tfh differentiation occur in late effector stages. Tfh10 cells were also enriched in aged individuals 7 days after vaccination; however, the low number of cells prevented statistical analyses.

The lack of differences in progenitor Tfh cells combined with the bifurcation of effector Tfh cells in young and aged individuals suggest age-related reprogramming occurs after initial Tfh differentiation. To understand the functional relevancy of age-related bifurcation of Tfh cells during late effector states, we assessed DEGs between Tfh Effector 1 (young-enriched) and Tfh Effector 2 (age-enriched) clusters. Despite both being identified as effector Tfh clusters, Tfh Effector 1 and 2 had substantial differences in gene expression (Figure 6D). Age-associated Tfh Effector 2 had increased expression of Tfh effector genes such as *BCL6*, *CXCR5*, *PDCD1*, *ICOS*, and *S1PR2* but also heightened expression of negative regulators of Tfh cells such as *ID2*, *KLF2*, and *BHLHE40*. In contrast, youth-associated Tfh Effector 1 had higher expression of positive regulators of initial Tfh differentiation such as *TCF7* and *LEF1*. These data, along with the higher pseudotime of Tfh Effector 2 (Figure 5F), suggest that Tfh Effector 2 is a heightened effector state that originates from Tfh Effector 1 cells. In addition, Tfh Effector 2 had higher expression of cellular stress/senescence genes, such as *CCL5*, *CCL20*, *TNFAIP3*, *FOS*, *DUSP1*, and *ATF4*, suggesting the heightened effector Tfh state in aging is accompanied by stress/senescence, similar to our findings in mouse models. To further assess differences between Tfh Effector 1 and 2 clusters, we conducted Gene Set Enrichment Analysis (GSEA). The top gene sets in Tfh Effector 1 were oxidative phosphorylation and fatty acid metabolism, suggesting heightened metabolic states (Figure 6E). In contrast, TNF-α and IL-2/Stat5 signaling were enriched in Tfh Effector 2, suggesting cytokine-mediated changes (Figure 6E). Unfortunately, age-dependent changes within Tfh Effector 1 and Effector 2 clusters individually could not be interrogated because of the lack of statistical power.

To understand clonal dynamics of circulating Tfh cells after vaccination, we assessed TCR sequences from the scRNA-Seq dataset. We evaluated the Shannon index (as a measure of clonal evenness) and the Chao1 index (as a measure of clonal diversity) and found both were similar at baseline between aged and young individuals but progressively diverged, with a lower Shannon index and higher Chao1 index in aged individuals at day 14 after vaccination (Supplemental Figure 9D). This combination indicates a more polyclonal population in aging but that some clones can substantially expand. However, the power of these calculations is suboptimal because of limited clonal expansion in most circulating Tfh cells. The most expanded Tfh clones were found in the Tfh Cytotoxic cluster as well as in the Tfh10 cluster (Figure 6F). We next assessed clonal sharing between Tfh clusters. We found very few clones shared between Tfr and Tfh cells, consistent with Tfr cells originating from natural Treg precursors and not naive or Tfh cells (Figure 6G). However, we found some clonal sharing between Tfh cell clusters, including between progenitor Tfh clusters (Tfh Prog 1, Tfh Prog 2) as well as effector clusters (Tfh Effector 1, Tfh Effector 2, Tfh10, Tfh Cytotoxic), suggesting the same clones can differentiate progressively through these Tfh differentiation stages. There was no clear difference in clonal sharing patterns between young and aged expanded clones (Figure 6G). Together, these data indicate that Tfh cells undergo progressive differentiation after human influenza vaccination and that settings of aging rewire late effector stages to induce a hyper effector Tfh state (Figure 6H).

### The inflammaging microenvironment reprograms late effector Tfh cell differentiation.

**Figure 7:**
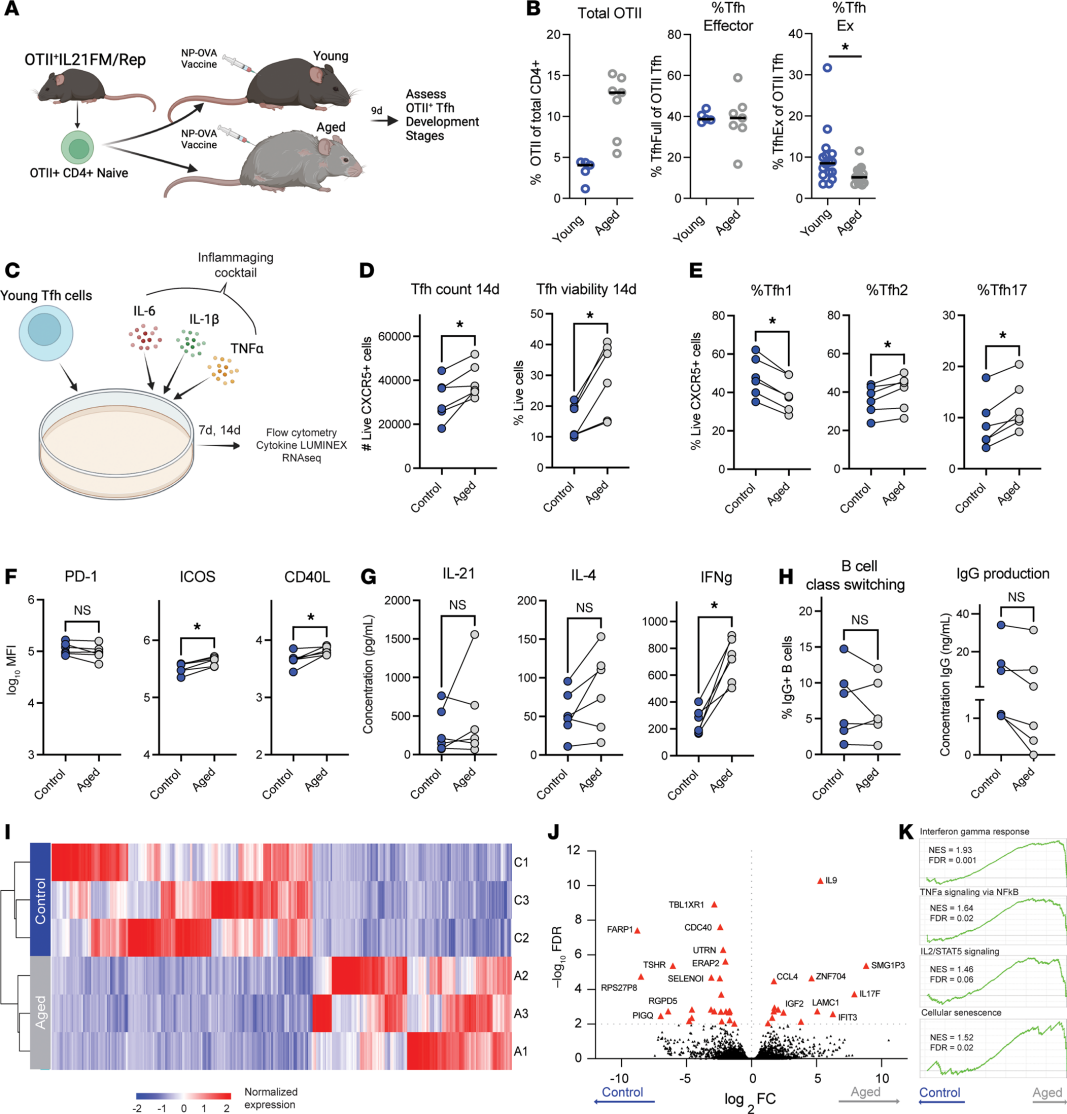
The inflammaging microenvironment rewires Tfh cell differentiation to a hyperinflammatory and senescent-like transcriptional state. (**A**) Experimental schematic to assess effects of the aged microenvironment on Tfh differentiation in vivo. Total splenic CD4^+^ T cells from OT-II^+^IL21FM/Rep (OT-II^+^Tg.*Il21*^Cre^*Rosa26*^LoxSTOPLoxTdTomato^*Il21*^VFP^) were adoptively transferred to 8-week-old (young) or 80-week-old (aged) mice, which were given an NP-OVA vaccine. (**B**) The frequency of dLN OT-II^+^ cells from the total CD4^+^ population (left) and of fully differentiated effector Tfh (middle) and TfhEx (right) from the total OT-II^+^ population. Each point represents an individual mouse. Results are reflective of 1 representative experiment (left, middle) or concatenated data from 2 experiments (right). (**C**) Experimental schematic to assess effects of the inflammaging microenvironment on human late effector Tfh differentiation in vitro. CD4^+^CXCR5^+^ Tfh cells sorted from healthy donors were incubated with an inflammaging cocktail containing 10 ng/mL each of IL-6, IL-1β, and TNF-α. (**D**) Tfh cell count (left) and viability (right) by spectral flow cytometry. (**E**) Tfh cell polarization to Tfh1 (left; CXCR3^+^CCR6^–^), Tfh2 (middle; CXCR3^–^CCR6^–^), or Tfh17 (right; CXCR3^–^CCR6^+^). (**F**) Expression of Tfh activation markers PD-1 (left), ICOS (middle), and CD40L (right). (**G**) Concentration of IL-21 (left), IL-4 (middle), and IFN-γ (right) in culture supernatants. (**H**) (Left) Frequency of class-switched B cells from cultures as in **C**. (Right) Concentration of IgG in culture supernatant in experiments. (**I**) Heatmap of genes in Tfh cells cultured in control (blue) or inflammaging (gray) conditions assessed 7 days after culture. (**J**) Volcano plot of *P* value and fold-change for genes in Tfh cells (red triangles *P* < 0.01; fold-change > 2). (**K**) GSEA of selected Hallmark gene modules and senescence gene module (29) in Tfh cells from **I**. All displayed data points represent mean value of technical replicates. *P < 0.05, uncorrected Mann-Whitney *U* test or Wilcoxon’s signed-rank test.

Settings of aging have been associated with increases in some pro-inflammatory cytokines in the circulation or tissues, commonly referred to as an inflammaging microenvironment. Moreover, age-related transcriptional rewiring of late effector stages in our data indicated elevated cytokine signaling, including for TNF-α, which was previously shown to be a component of inflammaging (42, 43). Therefore, we next investigated whether exposure to age-associated environments is sufficient to rewire late effector Tfh cells. To assess this in murine settings, we utilized an adoptive transfer system in which the differentiation stage of Tfh cells can be identified through fate mapping and direct reporting of the cytokine IL-21 (14). OT-II^+^CD4^+^ T cells were isolated from OT-II^+^Tg.*Il21*^Cre^*Rosa26*^LoxSTOPLoxTdTomato^*Il21*^VFP^ (OT-II^+^IL21FM/Rep) mice and adoptively transferred to 8-week-old (young) or (aged) 80-week-old mice that received a vaccine containing 4-hydroxy-3-nitro-phenylacetyl hapten–OVA (NP-OVA) (Figure 7A). After 9 days, adoptively transferred OT-II^+^ Tfh cells were identified (gated as Va2^+^Vb5^+^CD4^+^CD19^–^CXCR5^+^) and assessed for developmental stage by past/current expression of IL-21, as published previously (14). The frequency of total OT-II^+^ cells was higher in aged recipients (Figure 7B). Moreover, the frequency of fully differentiated effector Tfh cells (identified as Va2^+^Vb5^+^CD4^+^CD19^–^CXCR5^+^TdTomato^+^VFP^+^) was indistinguishable between young and aged recipients. However, the frequency of Tfh cells that downregulated the effector program by extinguishing IL-21 production into a resting stage (TfhEx; Va2^+^Vb5^+^CD4^+^CD19^–^CXCR5^+^TdTomato^+^VFP^–^) was lower in aged compared with young mice. These data indicate that in an aged microenvironment, Tfh cells from young mice can undergo early differentiation normally but have defects in later stages of Tfh differentiation where they are unable to transition away from effector states.

To understand if the inflammaging microenvironment could also reprogram later stages of Tfh development in human cells, we performed experiments in which partially differentiated Tfh cells were sorted (as live^+^CD4^+^CXCR5^+^) from young, healthy donors and underwent further differentiation in the presence of an artificial, inflammaging environment (Figure 7C). Cytokines in the inflammaging environment included IL-6, IL-1β, and TNF-α because these are higher in the peripheral circulation of aged humans (42, 43) as well as our finding that age-related Tfh Effector 2 cells have evidence of TNF-α signaling at the transcriptional level. Total cell counts and viability of Tfh cells were similar in inflammaging and control environments after 7 days in cell culture, indicating that this combination of cytokines is not directly toxic to cells (data not shown). However, after 14 days, we found increases in both cell count and viability in inflammaging versus control settings, suggesting that the inflammaging cocktail enhances proliferation or prolongs the lifespan of Tfh cells in vitro (Figure 7D). Moreover, we found a shift in the polarization of Tfh cells from a Tfh1 phenotype toward a Tfh2/Tfh17 phenotype in inflammaging settings (Figure 7E). Furthermore, in the inflammaging microenvironment, Tfh cells appeared more activated with enhanced surface expression of ICOS and CD40L, as well as enhanced production of IFN-γ (Figure 7G). However, there were no statistical differences in PD-1 expression or secretion of IL-21 or IL-4 (Figure 7, F and G). Furthermore, we tested the ability of Tfh cells to induce B cell differentiation in these assays. We added B cells (from the same donor) to cultures after 7 days and allowed these cells to differentiate for 7 more days. Despite increases in Tfh cell numbers as well as cytokine production in inflammaging settings, B cells underwent slightly reduced class switching and production of IgG; however, these did not reach statistical significance (Figure 7H). Together, these data support the hypothesis that the aged microenvironment alters Tfh programming.

To more completely understand how the inflammaging microenvironment controls late effector Tfh programs, we performed bulk RNA-Seq transcriptional analysis of Tfh cells cultured in inflammaging or control microenvironments. We found transcriptional changes in Tfh cells cultured in the inflammaging versus control microenvironments (Figure 7I). Genes that were upregulated in Tfh cells from the inflammaging microenvironment included *IL17F*, *IL9*, and the senescence gene *CCL4* (Figure 7J). Enhanced production of these cytokines was verified by ELISA of culture supernatants (data not shown). Last, we performed GSEA to assess broader pathways upregulated in Tfh cells because of the inflammaging microenvironment and found cytokine responses such as IFN-γ response, TNF-α signaling, and IL-2 signaling (Figure 7K). In addition, the inflammaging microenvironment enhanced expression of genes associated with cellular senescence. Taken together, these data suggest the inflammaging microenvironment may be sufficient to induce prolonged/heightened activation, proliferation, cytokine production, and cellular senescence-like programs in effector Tfh cells. Therefore, extrinsic factors in the aged microenvironment may rewire later effector stages of Tfh cells (Supplemental Figure 10).

## Discussion

An existing paradigm posits that immunity in aged individuals is an attenuated, yet mechanistically similar, version of a youthful immune system. Based on this, one current strategy to enhance vaccine responses in elderly individuals is the administration of an increased dose of a vaccine initially developed for young individuals (44). Although some high-dose vaccines do increase immunogenicity in older adults, it is unclear whether this is due to correcting underlying Tfh defects in aging or by partially circumventing these defects. An emerging paradigm posits that the aged immune system may have distinct cellular programming compared with the young immune system. If true, then therapeutic strategies to enhance immunity in older individuals need to specifically target age-related reprogramming. Tfh differentiation in the context of aging is poorly understood. Utilizing a combination of murine models and human samples, we identified that Tfh cells undergo progressive differentiation from a progenitor to effector state in response to seasonal influenza vaccination. Although Tfh differentiation is expected to occur predominantly in the dLN, both progenitor-like and late effector Tfh cells can be found in the circulation, where they exhibit diverse transcriptional states offering a glimpse into LN responses. In settings of advanced age, though early stages of Tfh differentiation occur normally, later Tfh differentiation stages become rewired to induce a hyperactivated, yet less functional, cellular state, which can be observed in both dLNs and blood. Mechanistically, we identified that the inflammaging microenvironment was sufficient to induce some age-related rewiring in effector Tfh cells. This rewiring included not only a hyperactivated state with enhanced production of cytokines and cellular senescence-like programs but also a defective ability to transition to post-effector resting states. These studies suggest that age-related defects in vaccine responsiveness are at least partially due to rewiring of later stages of Tfh differentiation and suggest strategies to enhance vaccine efficacy in aged individuals need to reverse age-related rewiring of Tfh differentiation.

We previously showed that Tfh cells undergo progressive differentiation in LNs (14). However, Tfh cells can be found in lymphoid organs as well as the circulation (15, 45), the latter of which is more easily accessible in human patients. How circulating Tfh cells reflect progressive differentiation in lymphoid organs remains enigmatic, particularly in the setting of seasonal influenza vaccination. In mice, we found evidence that progenitor-like Tfh cells transition to late effector-like Tfh cells in the dLN, which exhibit clonal overlap with expanded blood Tfh cells. Although expanded effector Tfh cells in dLNs were transcriptionally homogenous, clonally expanded cells found in the circulation had more diverse transcriptional programming. We hypothesize that upon gaining access the circulation, effector Tfh cells slowly downregulate their effector program, revealing transcriptional heterogeneity. Our human cTfh data verify the presence of progenitor-like and effector-like Tfh cells in the circulation. Importantly, effector-like Tfh cells increased in circulation after influenza vaccination, indicating that these cells likely originated from effector cells in the dLN.

Prior studies have demonstrated that Tfh cells exhibit phenotypic and transcriptional changes in aging and that these changes correlate with poor vaccine responsiveness (18, 21, 22, 28, 31, 32). Consistent with previous reports, our human data showed that ICOS^hi^CD38^hi^ cTfh cells increase after influenza vaccination (31, 32). Furthermore, our data suggest that the ICOS^hi^CD38^hi^ cTfh response is more substantial in young versus aged individuals. This observation was not found in previous reports and may be due to differences in cohort composition (31, 32). scRNA-Seq analysis of human cTfh following influenza vaccination revealed a similar progenitor-like state in both young and aged individuals, indicating normal early Tfh differentiation. However, we found a bifurcation in later effector stages with aged effector Tfh cells rewired with increased cytokine production/signaling and a cellular senescence-like transcriptional signature. Importantly, the ability of these cells to expand suggests the senescence-like transcriptional signature may not result in functional senescence. Collectively, we refer to these cells as age-rewired Tfh cells. The appearance of age-rewired Tfh cells is slightly distinct in mice and humans. In aged mice, we observe the accumulation of age-rewired Tfh cells with a *Cxcr3*^+^*Il21*^+^*Ifng*^+^ transcriptional profile. Furthermore, the murine cell population is clonally linked to a Tfh population with a cellular senescence gene signature, suggesting that these cells progressively increase the degree of age-rewiring over time. Unfortunately, we did not collect a prevaccination sample to discern whether some preexisting Tfh cells may have contributed to the data. In aged humans, we observe age-rewired Tfh cells with TNF-α and IL2/Stat5 transcriptional signatures, accompanied by reciprocal downregulation of metabolic pathways, such as oxidative phosphorylation and fatty acid metabolism.

One hypothesis for the origin of age-reprogrammed cells is that the inflammaging microenvironment induces rewiring of the Tfh developmental program such that effector Tfh cells persist in a hyperactivated state. The addition of known inflammaging cytokines, TNF-α, IL-1β, and IL-6 (42), was sufficient to recapitulate some features of prolonged cellular survival, hyperactivation, cytokine production, and cellular senescence programs. We have previously described a post-effector Tfh resting state, termed TfhEx cells, that are epigenetically poised and can return to the effector state (14). In this study, we use similar strategies to identify TfhEx cells in the context of aging and find that Tfh cells undergo normal early differentiation but during the effector stage have a reduced ability to convert to the resting TfhEx state. We hypothesize that transition between the effector and post-effector resting TfhEx stage is required to prevent hyperactivation/dysfunction, thereby maintaining a balanced effector population over time. We also hypothesize that the inflammaging microenvironment prevents this transition, and the age-rewired phenotype is a consequence of a sustained effector state.

Notably, this paradigm of a bifurcation in Tfh development with accumulating hyperactivation or senescence programs accompanied by limited TfhEx conversion is analogous to age-related changes observed in non-Tfh CD4^+^ as well as CD8^+^ T lymphocyte populations. It is likely that the TfhEx state represents a quiescent, memory-like phenotype (14). While memory Tfh populations have been observed by multiple groups, factors controlling them remain enigmatic (46–48). One hallmark of aged T cells is a reduction in the more quiescent central memory T cell pool in favor of a larger effector pool (49, 50). Therefore, we hypothesize a more global rewired transcriptional program occurs in aged T cells, which is driven by the inflammaging microenvironment.

Our data suggest that current strategies to overcome age-related defects in vaccine responsiveness by increasing antigen dose or adjuvant activity will not necessarily address the underlying age-rewired Tfh cell phenomenon and may even exacerbate it. Indeed, most aged participants in our study received a high-dose influenza vaccine, yet we identified age-related Tfh transcriptional changes. An alternative strategy to prevent the age-rewired state and allow proper cycling through post-effector quiescent/memory states in Tfh cells is likely required to reverse the altered humoral immunity observed in aging. However, additional studies are required to uncover the precise ways to deprogram defects in late effector Tfh cells. Moreover, any strategy to durably overcome age-rewired Tfh cell differentiation and restore a young Tfh cell phenotype would need to be integrated with other targeted immune manipulations to reverse the underlying inflammaging environment. While complex and multifaceted, the reversal of age-related Tfh rewiring has the potential to enhance vaccine-elicited responses in the aged population and thereby to improve the health and longevity of this vulnerable population.

## Methods

### Sex as a biological variable.

Male mice were used for murine aging studies. Both male and female human participants were included in this study. Findings are expected to apply to both sexes.

### Mice.

Young (8 ± 2 weeks) or aged (~80 ± 2 weeks) male C57BL/6 mice were obtained from Jackson Laboratory or the NIH National Institute of Aging. *Rosa26*^Lox-STOP-Lox-TdTomato^, *Il21*^VFP^, and OT-II were purchased from Jackson Laboratory. Tg.*Il21*^Cre^ were from Uta Hoepken (Max-Delbrück Center for Molecular Medicine, Berlin, Germany) and have been published (51). For vaccination studies, mice were administered an unadjuvanted Afluria influenza vaccine (1.5 μg HA protein) and tissues were harvested. For adoptive transfers, 1 × 10^6^ splenic CD4^+^ T cells from OT-II^+^ IL-21^FM/Rep^ (Tg.*Il21*^cre^*Rosa26*^Lox-STOP-Lox-TdTomato^*Il21*^VFP^) mice were transferred to young or aged mice that received 100 μg NP-OVA (Biosearch Technologies) mixed with Addavax adjuvant (InvivoGen). All mice used in the experiments were kept in normal housing conditions that included 12-hour light/12-hour dark cycle, at 22°C and 42% humidity, and were fed 5053 PicoLab diet 20 (LabDiet).

### Human participants.

Influenza vaccinee samples were collected during the 2022–2023 influenza vaccine season. Study exclusion criteria included history of allergic reaction to influenza vaccination or eggs, Guillain-Barre Syndrome, receipt of viral vaccines within 1 month of study initiation, any ongoing infections, and pregnancy. Young donors were between 18 and 38 years of age, and older donors were above 65 years of age. Donors designated their race and ethnicity at the time of study enrollment (Supplemental Table 3). Participants received the 2022–2023 influenza vaccine formulation (A/Wisconsin/588/2019, A/Darwin/6/2021, B/Austria/1259417/2021, and B/Phuket/2073/2013). Day 0 blood draws were collected before vaccination, and postvaccination samples were collected at day 7 ± 1, day 14 ± 1, and day 30 ± 3.

### Plasma and PBMC samples.

Blood was collected in K2 EDTA Vacutainer Tubes (BD 366643) and stored at 4°C until processing. Samples were diluted at a 1:1 with PBS containing EDTA (Miltenyi Biotec 130-091-222) and 2% v/v heat-inactivated fetal bovine serum (FBS) (Gibco A52567-01) and layered on Ficoll-Paque PLUS (Cytiva 17144003). Samples were centrifuged 20 minutes at 400*g* at room temperature. Plasma was then collected as the top layer and frozen at –20°C for serological analyses, and the PBMC layer was collected, subjected to ACK (Lonza BP10-548E) lysis, and cryopreserved in Bambanker (GC Lymphotec 302-14681) freezing in a 1°C freezing container (Nalgene 5100-0001) at –80°C overnight before transfer to storage in liquid nitrogen.

### High-parameter spectral flow cytometry.

Cryopreserved PBMCs were rapidly warmed and added drop-wise to human growth media (RPMI 1640 containing 10% FBS, 10 mM HEPES, 100 U/mL penicillin, and 100 μg/mL streptomycin) in a 15 mL tube (Falcon, Corning), then resuspended in 200 μL of cell staining buffer. Cells were washed once with cell staining buffer (Gibco Dulbecco’s PBS containing 1% FBS and 4 mM HEPES) and stained with ViaDye Violet (Cytek Biosciences) at 1:1,000 dilution for 20 minutes. After 2 washes, a primary antibody cocktail was added (from BD Biosciences except where noted) for surface marker staining at 4°C for 60 minutes. The antibodies for human Tfh profiling included anti-CD3 (UCHT1, BUV496), anti-CD4 (M-T477, BUV395), anti-CD8 (RPAT8, BV510), anti-CD14 (M5E2, BV510), anti-CD19 (BioLegend H1B19, BV510), anti-CD45RA (HI100, AF700), anti-ICOS (BioLegend C398.4A, PE-Cy7), anti-CD226 (DX11, BB700), anti-CCR7 (3D12, BV650), anti-CXCR3 (1C6, BV480), anti-CCR6 (11A9, BUV737), anti-CXCR5 (552118, Biotin), anti–PD-1 (BioLegend EH12.287, BV785), anti-Tigit (BioLegend A15153G, APC-Cy7), and anti-CD38 (BioLegend HIT2, AF488). Additional antibodies for human in vitro culture included anti-CD40L (BioLegend 24-31, AF700), anti-CD69 (555532, PE-Cy5), anti-CD25 (555431, FITC), anti-Ox40 (ACT35, BUV496), anti-CD19 (SJ25C21, APC-Cy7), and anti-GL7 (BioLegend GL7, PB). Following washes, cells were stained with streptavidin-BV421 (BioLegend 405225) at 4°C for 30 minutes. Cells were fixed and permeabilized using Cell Fixation/Permeabilization Kit (Invitrogen). For intracellular staining, anti-BCL6 (K11291, PE) and anti-FoxP3 (236AE7, AF647) were added to cells at 4°C for 60 minutes. In vitro experiment B cells were additionally stained with anti-IgG Fc (BioLegend M1310G05, PE). Staining of murine dLNs and PBMCs was performed as described (14). Data were acquired on a Cytek Aurora (5-laser configuration), and data were analyzed with FlowJo version 10.

### Cell sorting.

Human cryopreserved PBMCs were reanimated as above. For both mouse and human samples, cells were magnetically enriched for CD4 by positive selection (Miltenyi Biotec) and stained as above. Antibodies included anti-CD4 (M-T477, BUV395), anti-CD19 (SJ25C21, APC-Cy7), and anti-CXCR5 (552118, Biotin). Following washing, cells were stained with streptavidin-BV421 (BioLegend 405225) at 4°C for 30 minutes. Then 7-AAD Live/Dead dye (Thermo Fisher Scientific) was added at 1:1,000. Staining of murine dLNs and PBMCs was performed as described (14). Stained cells were sorted on a BD FACSAria II cell sorter (85 μm nozzle) as CD4^+^CD19^–^CXCR5^+^ cells.

### scRNA-Seq and quality control.

Sorted cells were stained using TotalSeq-C antibodies from BioLegend (clones LNH-94 and 2M2). Cells were loaded onto a Chromium chip K and encapsulated using the 5′ kit V2 (10x Genomics). Subsequently, cDNA synthesis and library preparation were executed as per the manufacturer’s instructions, and cDNA were sequenced on an Illumina NovaSeq. Postsequencing reads underwent processing using Cell Ranger (10x Genomics), and quantification was performed with STAR aligner. Output data were imported into the Seurat package for further analyses.

### Single-cell RNA data processing and analysis.

Initial processing steps included demultiplexing and doublet exclusion with HTODemux function. Filtering based on unique molecular identifier (UMI) count, gene count, log-transformed genes per UMI, and mitochondrial RNA content was performed. For mouse data, normalization and variance stabilization were performed using the SCTransform function (v.2) based on the 3,000 most variable genes, while regressing cell cycle phase, mitochondrial, and ribosomal mapping. For human data, normalization and variance stabilization were performed via the Seurat v5 pipeline (NormalizeData, FindVariableFeatures, and ScaleData). Identity-based filtering was applied after comparing each cell with the Immunologic Genome Project dataset via the SingleR (mouse) or Azimuth (human) pipeline to exclude contaminating cells. Mouse Tfr cells were defined by expression of *Foxp3* or SingleR as a Treg-dominant transcriptional profile. Dimensionality reduction was accomplished using UMAP, with exclusion of TCR-related genes. Differential gene expression analysis between multiplexed samples was conducted using the DESeq2 model within the Seurat FindAllMarkers command. Module scores were computed using the AddModuleScore function. Milo neighborhood analysis was completed using the miloR package (v1.6.0). TCR analyses were completed using scRepertoire package (v2.0), with clones defined as an identical amino acid sequence. Pseudotime analysis was completed using the Monocle3 package (v1.3.7).

### MSD binding.

Standard binding 384-well MSD plates (MSD LA21XA-4) were coated overnight with Influenza A/Wisconsin/588/2019 H1 HA in PBS at 1 μg/mL. Plates were washed 3 times with PBS + 0.05% Tween 20 (washing buffer), blocked for 1 hour with 3% w/v instant nonfat dry milk (Foodhold USA) (blocking buffer), washed 3 times, incubated with plasma samples diluted 1,000× in blocking buffer for 2 hours, washed 3 times, incubated with Sulfo-TAG–labeled anti-human antibody (Goat) (MSD R32AJ-1), diluted to 1 μg/mL in blocking buffer for 1 hour, washed 6 times, and read with 1× read buffer (MSD R92TC) on an MSD Sector S 600. All values are reported as the mean of 3 technical replicates.

### HAI titer.

HAI titers were determined as previously described (52). For H1 HAI, Influenza A/Wisconsin/588/2019 was amplified in MDCK cells (ATCC, CCL-34), and HAI titers were determined using 0.5% turkey red blood cells (Innovative Research ITKRBC5P) diluted in PBS. Samples were measured in duplicate, and titers are reported as log_2_ inhibition titers.

### Tfh in vitro cell culture.

A total of 5 × 10^4^ human Tfh cells from samples above were sorted and added to media (RPMI 1640 containing 10% FBS, 10 mM HEPES, 100 U/mL penicillin, and 100 μg/mL streptomycin) in a U-bottom cell culture plate (Corning). Each well was supplemented with 6 × 10^4^ anti-CD3/anti-CD28 Dynabeads (Gibco). To simulate aged microenvironment, 10 ng/mL of IL-6, IL-1β, and TNF-α were added. Cells and supernatant were harvested after 7 days of culture. For B cell experiments, CD19^+^ cells were purified from PBMCs according to the manufacturer protocol (Miltenyi Biotec). Tfh cells were washed and 2 × 10^5^ CD19^+^ B cells were added to each well supplemented with anti-IgM (Jackson ImmunoResearch, 5 μg/mL). B and Tfh cells were harvested after an additional 7 days. Concentration of cytokines in the supernatant was measured by multiplex ELISA using Human ProcartaPlex Simplex kits (Thermo Fisher Scientific).

### Bulk RNA-Seq.

Bulk RNA-Seq was performed as previously published (14). In short, 5 × 10^4^ Tfh cells from in vitro cell culture experiments were harvested and resuspended in RLT buffer. RNA extraction was done utilizing MyOne Silane Dynabeads (Thermo Fisher Scientific). Fragmentation and barcoding using 8 bp barcodes and standard Illumina adaptors was performed. Agencourt AMPure XP bead cleanup (Beckman Coulter) was performed followed by 15 cycles of PCR amplification. Libraries underwent gel purification and quantification. Sequencing was carried out on an Illumina NextSeq sequencer (Illumina) with single-end 50 bp reads. Sequencing reads were aligned to the hg38 genome (Ensembl GRCh38.99) using the STAR aligner, followed by quantification using Salmon within the bcbio-nextgen 1.2.9 bulk RNA-Seq pipeline. The aligned and quantified data were processed using the bcbioRNAseq function. Differential gene expression analysis was subsequently conducted using the DESeq2 package in R.

### Statistics.

Statistical graphs and descriptive measures (such as frequency, percentage, mean, SD, median, and IQR) were used to summarize data. The Mann-Whitney *U* test was used to assess differences in study variables between groups. *P* values were not corrected for multiple comparisons. Correlation analyses using Spearman’s rank correlation were used to examine bivariate associations. Longitudinal modeling of repeated measures, using nonlinear mixed models, was used to examine and compare the patterns of overtime changes in immunologic and serologic variables. All *P* values are 2 sided, and a *P* value of less than 0.05 was considered significant. Statistical analyses were performed using R software version 4.3.1 and SAS software version 9.4 (SAS Institute).

### Study approval.

All mouse protocols used in this study were approved by the Brigham and Women’s Hospital Institutional Animal Care and Use Committee and the NIH *Guide for the Care and Use of Laboratory Animals* (National Academies Press, 2011). Influenza vaccinee samples were collected under a protocol approved by the Dana Farber Cancer Institute Institutional Review Board. All participants provided voluntary and informed consent for study participation. Additionally, all participants were compensated for their time and participation in the study.

### Data availability.

Both mouse and human scRNA-Seq as well as in vitro bulk RNA-Seq data sets are available on the NCBI GEO repository under accession number GSE286979. Data used for Figures 4 and 7 and for Supplemental Figures 2 and 8 are available in the Supporting Data Values file.

## Author contributions

CSN and MAP designed and performed experiments, acquired and analyzed data, and wrote the manuscript. JML, CJB, and CBC performed experiments and analyzed data. MGG, AS, PC, EB, and SR performed experiments. MG analyzed data and provided guidance on data analyses. PGM, MRC, HK, SGT, RA, MCH, and WAM provided key technical help. PTS designed experiments, analyzed data, wrote the manuscript, and secured funding. All authors edited the manuscript.

## Supplementary Material

Supplemental data

Supporting data values

## Version 1 03/04/2025

In-Press Preview

## Version 2 04/22/2025

Electronic publication

## References

[1] Murasko D (2002). Role of humoral and cell-mediated immunity in protection from influenza disease after immunization of healthy elderly. Exp Gerontol.

[2] Sasaki S (2011). Limited efficacy of inactivated influenza vaccine in elderly individuals is associated with decreased production of vaccine-specific antibodies. J Clin Invest.

[3] Müller L (2021). Age-dependent immune response to the Biontech/Pfizer BNT162b2 coronavirus disease 2019 vaccination. Clin Infect Dis.

[4] Wagner A (2018). Age-related differences in humoral and cellular immune responses after primary immunisation: indications for stratified vaccination schedules. Sci Rep.

[5] Weinberger B (2010). Decreased antibody titers and booster responses in tick-borne encephalitis vaccinees aged 50–90 years. Vaccine.

[6] Hainz U (2005). Insufficient protection for healthy elderly adults by tetanus and TBE vaccines. Vaccine.

[7] Burns EA (1993). Specific humoral immunity in the elderly: in vivo and in vitro response to vaccination. J Gerontol.

[8] Goronzy JJ, Weyand CM (2017). Successful and maladaptive T cell aging. Immunity.

[9] Linton PJ (2005). Vitamin E and immune response in the aged: molecular mechanisms and clinical implications. Immunol Rev.

[10] Lefebvre JS (2012). The aged microenvironment contributes to the age-related functional defects of CD4 T cells in mice. Aging Cell.

[11] Belongia EA (2015). Waning vaccine protection against influenza A (H3N2) illness in children and older adults during a single season. Vaccine.

[12] Crotty S (2019). T follicular helper cell biology: a decade of discovery and diseases. Immunity.

[13] Vinuesa CG (2016). Follicular helper T cells. Annu Rev Immunol.

[14] Podestà MA (2023). Stepwise differentiation of follicular helper T cells reveals distinct developmental and functional states. Nat Commun.

[15] Sage PT (2014). Circulating T follicular regulatory and helper cells have memory-like properties. J Clin Invest.

[16] Zhou P (2023). Longitudinal analysis of memory Tfh cells and antibody response following CoronaVac vaccination. JCI Insight.

[17] Bentebibel S-E (2016). ICOS(+)PD-1(+)CXCR3(+) T follicular helper cells contribute to the generation of high-avidity antibodies following influenza vaccination. Sci Rep.

[18] Herati RS (2014). Circulating CXCR5+PD-1+ response predicts influenza vaccine antibody responses in young adults but not elderly adults. J Immunol.

[19] Cavazzoni CB (2022). Follicular T cells optimize the germinal center response to SARS-CoV-2 protein vaccination in mice. Cell Reports.

[20] Lee JL, Linterman MA (2022). Mechanisms underpinning poor antibody responses to vaccines in ageing. Immunol Lett.

[21] Hill DL (2021). Impaired HA-specific T follicular helper cell and antibody responses to influenza vaccination are linked to inflammation in humans. Elife.

[22] Silva-Cayetano A (2023). Spatial dysregulation of T follicular helper cells impairs vaccine responses in aging. Nat Immunol.

[23] Zhou M (2014). The effect of aging on the frequency, phenotype and cytokine production of human blood CD4 + CXCR5 + T follicular helper cells: comparison of aged and young subjects. Immun Ageing.

[24] Agrawal A (2012). Increased IL-21 secretion by aged CD4+T cells is associated with prolonged STAT-4 activation and CMV seropositivity. Aging (Albany NY).

[25] Hou S (2019). FoxP3 and Ezh2 regulate Tfr cell suppressive function and transcriptional program. J Exp Med.

[26] Aloulou M (2016). Follicular regulatory T cells can be specific for the immunizing antigen and derive from naive T cells. Nat Commun.

[27] Maceiras AR (2017). T follicular helper and T follicular regulatory cells have different TCR specificity. Nat Commun.

[28] Sage Peter T (2015). Defective TFH cell function and increased TFR cells contribute to defective antibody production in aging. Cell Rep.

[29] Saul D (2022). A new gene set identifies senescent cells and predicts senescence-associated pathways across tissues. Nat Commun.

[30] Linterman MA, Hill DL (2016). Can follicular helper T cells be targeted to improve vaccine efficacy?. F1000Res.

[31] Herati RS (2021). Vaccine-induced ICOS^+^CD38^+^ circulating Tfh are sensitive biosensors of age-related changes in inflammatory pathways. Cell Rep Med.

[32] Herati RS (2017). Successive annual influenza vaccination induces a recurrent oligoclonotypic memory response in circulating T follicular helper cells. Sci Immunol.

[33] Zhu F (2023). Spatiotemporal resolution of germinal center Tfh cell differentiation and divergence from central memory CD4^+^ T cell fate. Nat Commun.

[34] Lindgren G (2017). Induction of robust B cell responses after influenza mRNA vaccination is accompanied by circulating hemagglutinin-specific ICOS^+^ PD-1^+^ CXCR3^+^ T follicular helper cells. Fronti Immunol.

[35] Olatunde AC (2021). Cytokine-skewed Tfh cells: functional consequences for B cell help. Trends Immunol.

[36] Shi J (2018). PD-1 Controls Follicular T Helper Cell Positioning and Function. Immunity.

[37] Sage PT (2018). Dendritic cell PD-L1 limits autoimmunity and follicular T cell differentiation and function. J Immunol.

[38] Almanan M (2020). IL-10–producing Tfh cells accumulate with age and link inflammation with age-related immune suppression. Sci Adv.

[39] Panwar B (2021). Multi–cell type gene coexpression network analysis reveals coordinated interferon response and cross–cell type correlations in systemic lupus erythematosus. Genome Rese.

[40] Zhang F (2019). Defining inflammatory cell states in rheumatoid arthritis joint synovial tissues by integrating single-cell transcriptomics and mass cytometry. Nat Immunol.

[41] Arazi A (2019). The immune cell landscape in kidneys of patients with lupus nephritis. Nat Immunol.

[42] Rea IM (2018). Age and age-related diseases: role of inflammation triggers and cytokines. Front Immunol.

[43] Batista MA (2020). Inflammaging in endemic areas for infectious diseases. Front Immunol.

[44] DiazGranados CA (2014). Efficacy of high-dose versus standard-dose influenza vaccine in older adults. N Engl J Med.

[45] Vella LA (2019). T follicular helper cells in human efferent lymph retain lymphoid characteristics. J Clin Invest.

[46] Feng H (2024). A novel memory-like Tfh cell subset is precursor to effector Tfh cells in recall immune responses. J Exp Med.

[47] Asrir A (2017). Interconnected subsets of memory follicular helper T cells have different effector functions. Nat Commun.

[48] Hale JS, Ahmed R (2015). Memory T follicular helper CD4 T cells. Front Immunol.

[49] Jain A (2023). Heterogeneity of memory T cells in aging. Front Immunol.

[50] Kim C (2018). Activation of miR-21-regulated pathways in immune aging selects against signatures characteristic of memory T cells. Cell Rep.

[51] Wichner K (2016). Dysregulated development of IL-17- and IL-21-expressing follicular helper T cells and increased germinal center formation in the absence of RORγt. FASEB J.

[52] Kaufmann L (2017). An optimized hemagglutination inhibition (HI) assay to quantify influenza-specific antibody titers. J Vis Exp.

