# Supplementary material for: The inflammaging microenvironment induces dysfunctional rewiring of Tfh cell differentiation
Source: JCI Insight. 2025 Mar 4;10(8):e187271. doi: 10.1172/jci.insight.187271 (PMC12016926; doi:10.1172/jci.insight.187271)
Supplement: Supplemental data [file jciinsight-10-187271-s008.pdf]

SUPPLEMENTAL MATERIAL

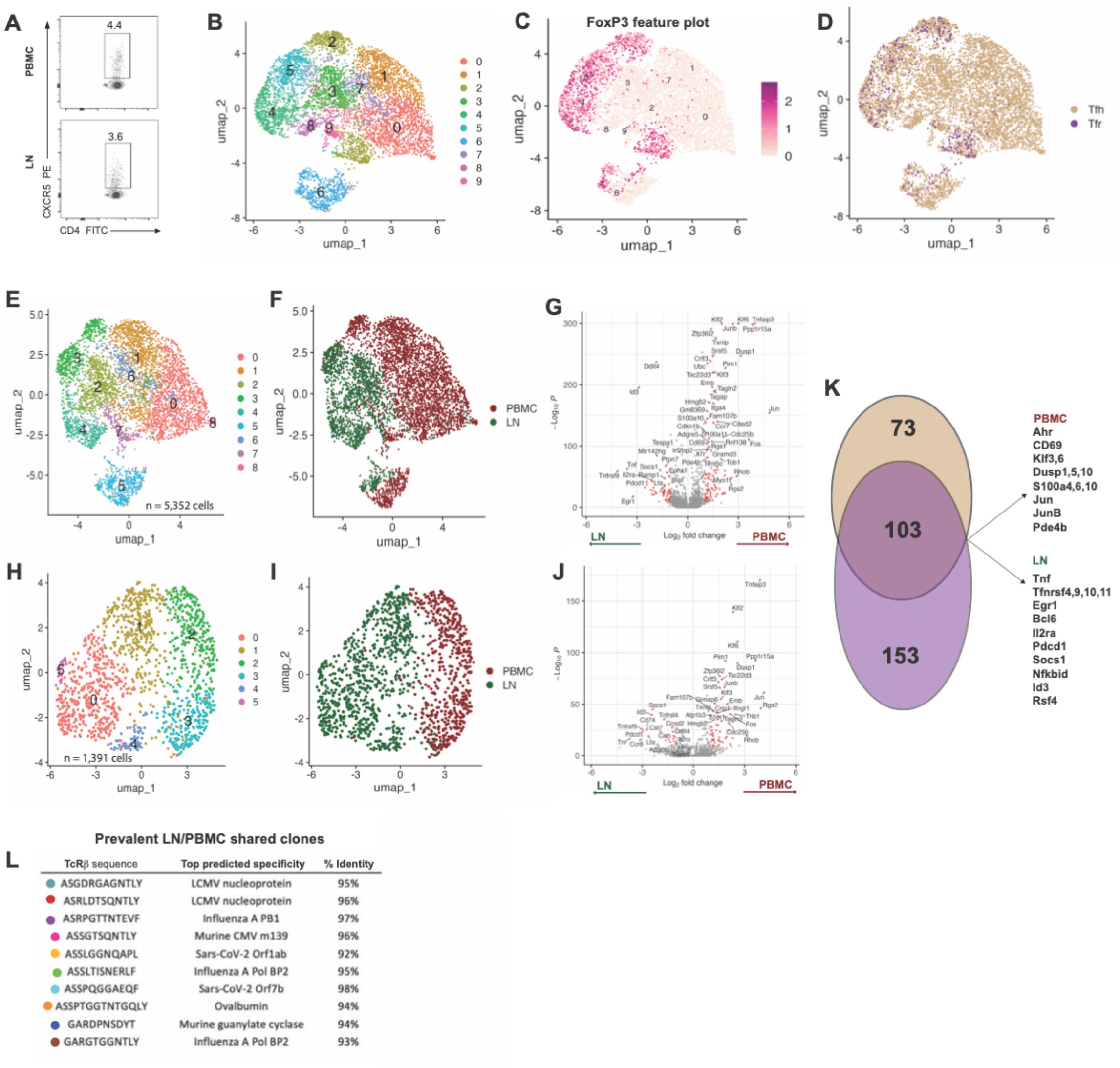

TcRβ sequence	Top predicted specificity	% Identity
ASGDRGAGNTLY	LCMV nucleoprotein	95%
ASRLDTSQNTLY	LCMV nucleoprotein	96%
ASRPGTTNTEVF	Influenza A PB1	97%
ASSGTSQNTLY	Murine CMV m139	96%
ASSLGGNQAPL	Sars-CoV-2 Orf1ab	92%
ASSLTISNERLF	Influenza A Pol BP2	95%
ASSPQGGAEQF	Sars-CoV-2 Orf7b	98%
ASSPTGGTNTGQLY	Ovalbumin	94%
GARDPNSDYT	Murine guanylate cyclase	94%
GARGTGGNTLY	Influenza A Pol BP2	93%

Supplemental Figure 1. Tfh and Tfr tissue-specific transcriptional profile after influenza vaccination. **(A)** Representative flow cytometry plots from $CD4^{+}CXCR5^{+}$ cell sorting, separated by tissue. Numbers denote $CXCR5^{+}$ percentage of total $CD4^{+}$ population. **(B-D)** UMAP projections of n=6,752 cells by unsupervised cluster assignment **(B)**, FoxP3 feature plot **(C)**, and by predicted cell type (assigned by SingleR) **(D)**. **(E and F)** UMAP projection of n=5,352 Tfh cells alone with unsupervised clustering **(E)** and separated by tissue **(F)**. **(G)** Volcano plot demonstrating differential gene expression (DEG) by tissue, with PBMC-enriched genes to the right of the y-axis and LN-enriched to the left. Red dots indicate genes meeting the significance threshold ($p < 10^{-10}$, fold change > 2). **(H and I)** UMAP projection of n = 1,391 Tfr cells alone with unsupervised clustering **(H)** and separated by tissue **(I)**. **(J)** Volcano plot demonstrating DEGs by tissue, with PBMC-enriched genes to the right of the y-axis and LN-enriched to to the left. Red dots indicate genes meeting the significance threshold ($p < 10^{-10}$, fold change > 2). **(K)** Venn diagram of PBMC vs. LN DEGs for Tfh (brown) and Tfr (purple). Select DEGs shared by Tfh and Tfr annotated to the right, separated by tissue. **(L)** TCR CDR3 amino acid sequence and top predicted specificity of the 10 most expanded clones shared between LN and blood Tfh. Data are from a single scRNAseq experiment of two individual mice concatenated.

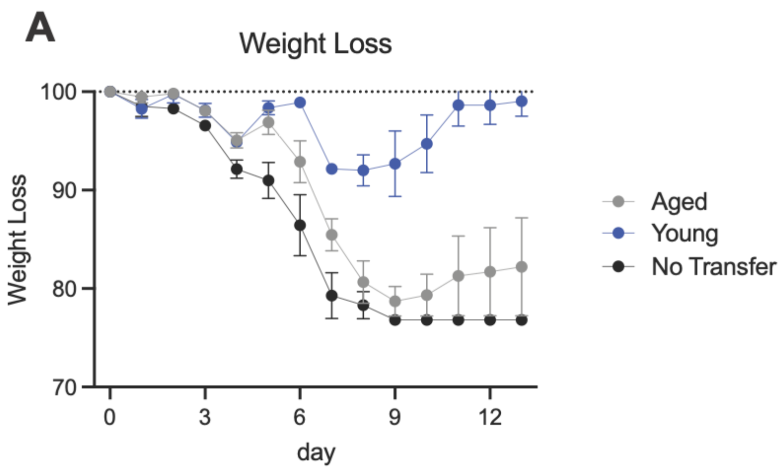

Supplemental Figure 2. Passive vaccination using serum from young, but not aged, mice is protective against severe influenza infection. Young mice were passively infused with 200ul serum from either aged or young inactivated H1N1(A/PR/8/34)/Addavax vaccinated donor mice (pooled from 3-4 mice). Recipients were challenged intranasally with $1LD_{50}$ of PR8. Daily weights were obtained for 14 days following infection. n=3 young, n=4 aged. Data points represent the mean weight, and bars SEM.

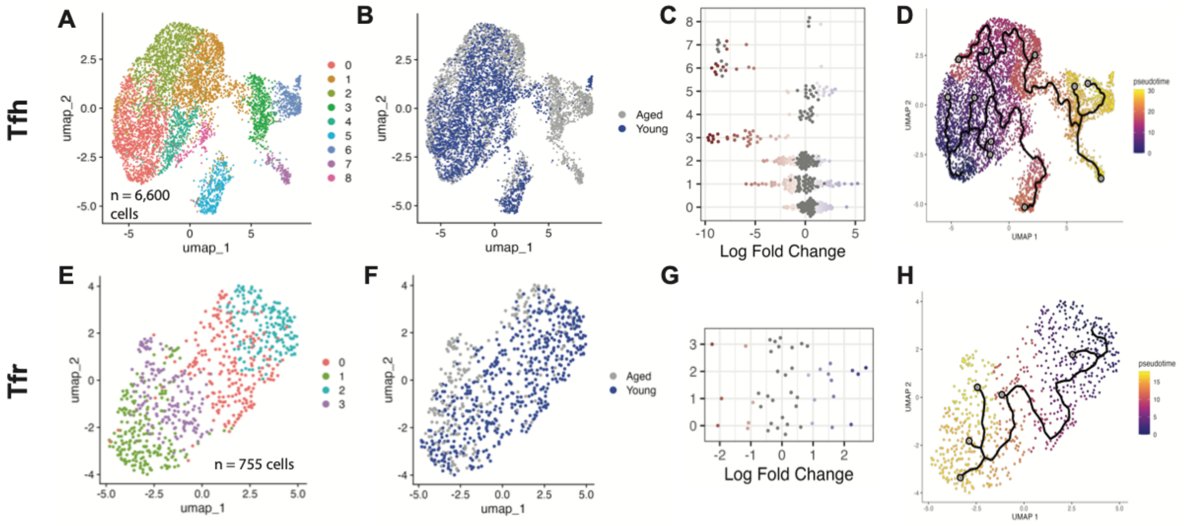

Supplemental Figure 3. Age-related transcriptional changes are more pronounced in cTfh cells than cTfr cells. (A and B) UMAP projections of n=6,600 young and aged cTfh by unsupervised cluster assignment **(A)** and age group **(B)**. (**C**) MiloR cell neighborhood analysis illustrating differential abundance of cTfh cell neighborhoods in young (blue) versus aged (red) mice, represented by individual cluster (FDR<0.05, FC>2). (**D**) UMAP projection of cTfh Monocle3 pseudotime trajectories. **(E and F)** UMAP projections of n=755 young and aged cTfr by unsupervised cluster assignment **(E)** and age group **(F)**. (**G**) MiloR cell neighborhood analysis illustrating differential abundance of cTfr cell neighborhoods in young versus aged mice, represented by individual cluster (FDR<0.05, FC>2). Data is concatenated data from two individual mice in each age group. (**H**) UMAP projection of cTfr Monocle3 pseudotime trajectories.

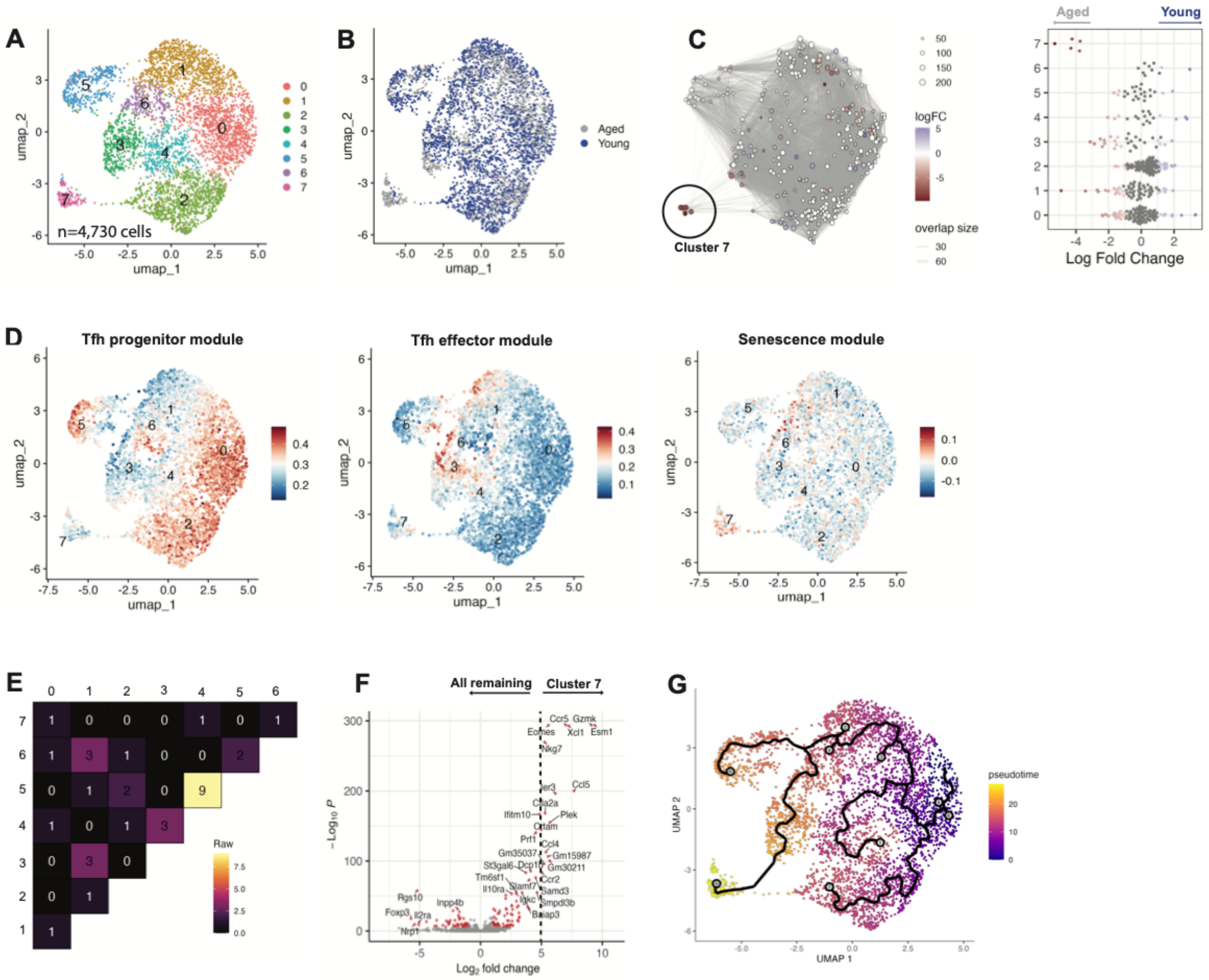

Supplemental Figure 4. Age-rewired Tfh cells with cellular senescence programs are also present in draining lymph node. (A and B) UMAP projections of n=4,730 young and aged lymph node follicular T cells by unsupervised cluster assignment **(A)** and age group **(B)**. **(C)** MiloR cell neighborhood analysis illustrating differential abundance of cell neighborhoods in young versus aged mice, represented in UMAP space or by individual cluster (FDR<0.05, FC>2). **(D)** Module score feature plots for a Tfh Progenitor module (14), Tfh Effector module (14), or a senescence module (29). **(E)** Clonal sharing (based on TCR sequence) including absolute number of shared clones between clusters. **(F)** Differentially expressed genes between cluster 7 and all remaining clusters. Red dots indicate ($p < 10^{-2}$, fold change > 2). (**G**) UMAP projection of lymph node follicular T cell Monocle3 pseudotime trajectories. Data is concatenated data from two individual mice in each age group.

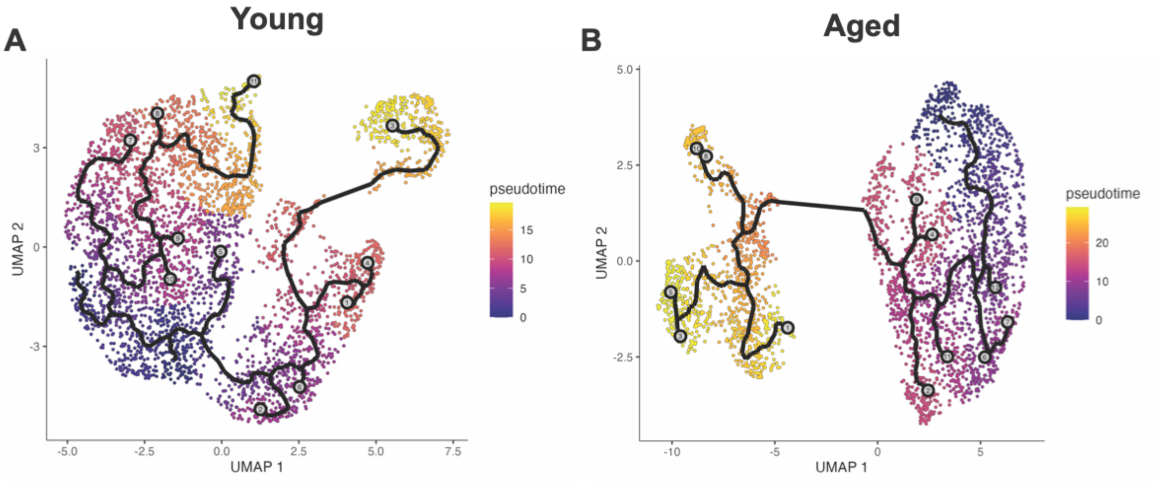

Supplemental Figure 5. Independent pseudotime analysis for young and aged mice. (**A and B**) UMAP projection of follicular T cell Monocle3 pseudotime trajectories for young (**A**) and aged vaccinated mice (**B**).

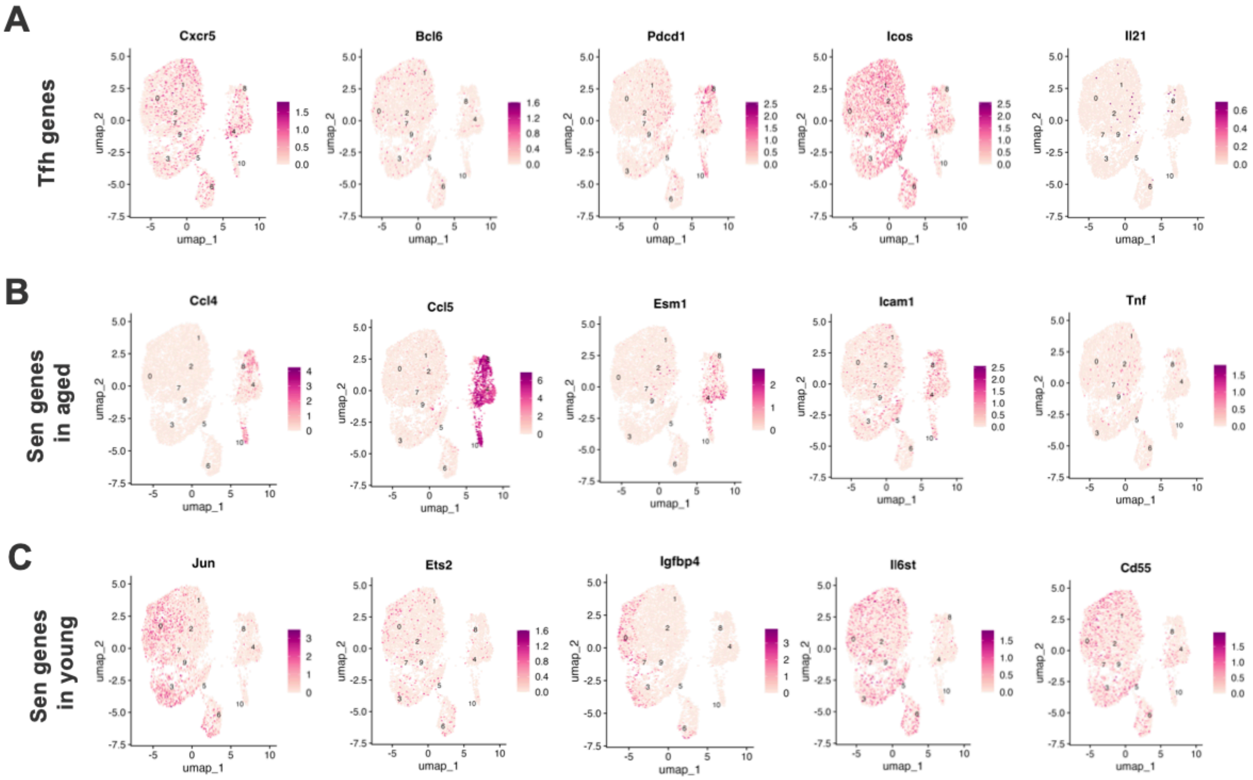

Supplemental Figure 6. Individual gene feature plots. (**A-C**) UMAP feature plot projections of n=6,752 cells denoting single gene expression for Tfh-associated genes (**A**), senescence-associated genes increased in aged cells (**B**), and senescence-associated genes increased in young cells (**C**).

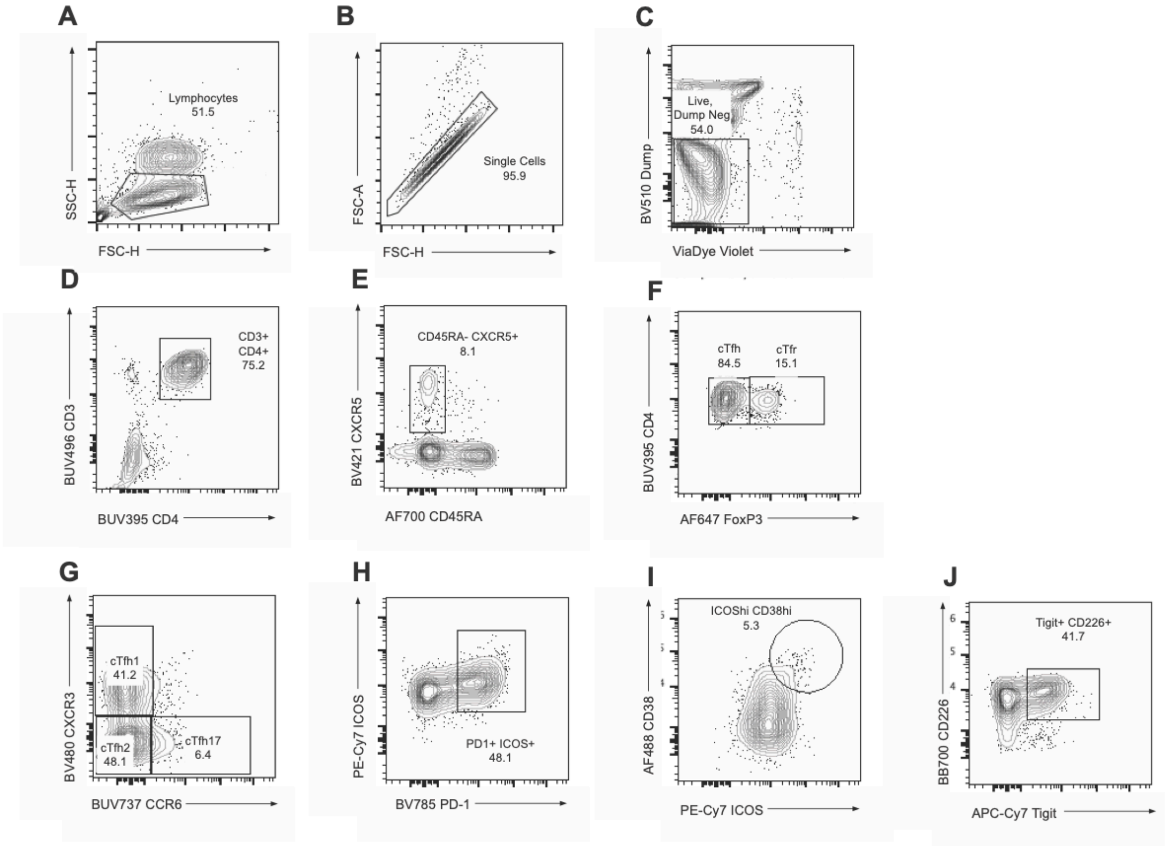

Supplemental Figure 7. High parameter spectral flow cytometry gating of follicular T cells. A lymphocyte gate was applied **(A)**, then FSC singlets **(B)**. Next, the live fraction of the "dump" negative ($CD8^-$ $CD14^-$ $CD19^-$) population was selected **(C)**, and subsequently the $CD3^+CD4^+$ subset identified **(D)**. Follicular T cells were defined as $CD45RA^-$ $CXCR5^+$ **(E)**, then cTfh and cTfr subpopulations distinguished by FoxP3 staining **(F)**. cTfh populations were further classified as $CXCR3^+CCR6^-$ (cTfh1), $CXCR3^-CCR6^-$ (cTfh2), or $CXCR3^-CCR6^+$ (cTfh17) **(G)**. Lastly, the fraction of cTfh co-expressing specific surface markers was defined including $PD1^+ICOS^+$ **(H)**, $CD38^+ICOS^+$ **(I)**, and $Tigit^+CD226^+$ **(J)**.

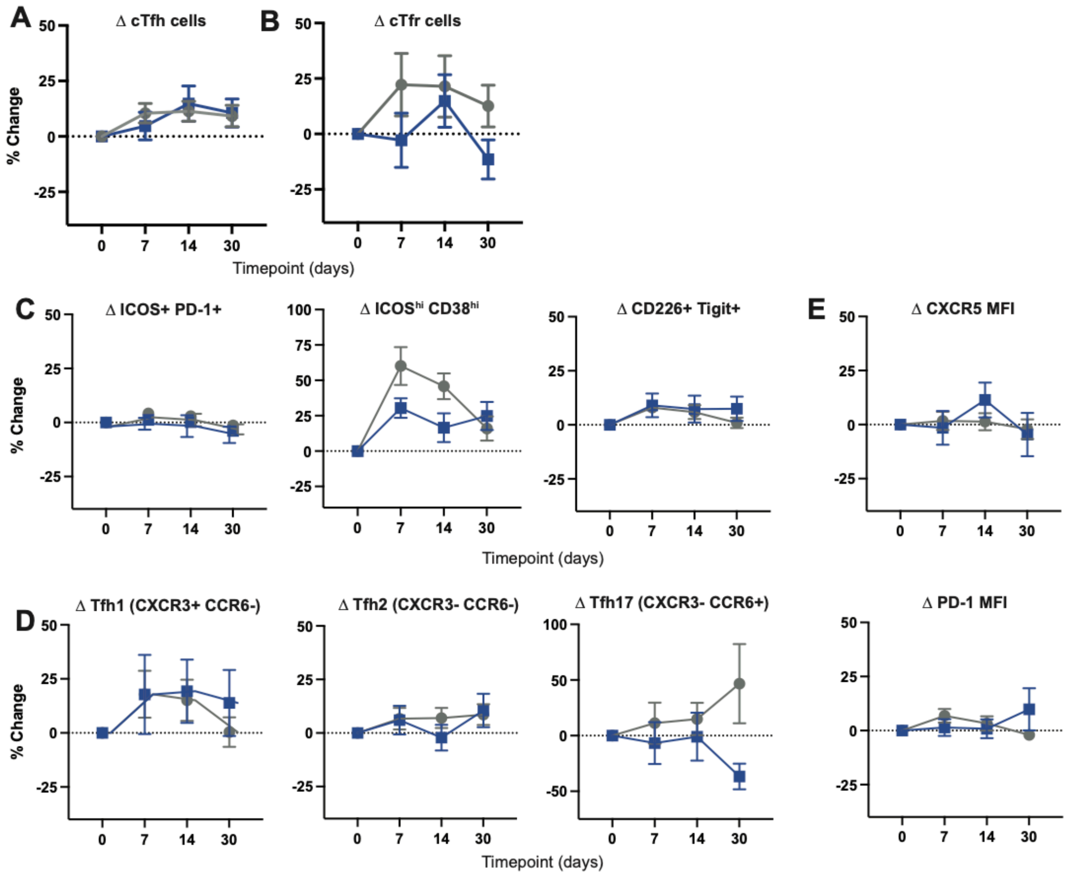

Supplemental Figure 8. Dynamic change of cTfh and cTfr populations after influenza vaccination. (A and B) Percent change from baseline (d0) of the cTfh frequency **(A)** and cTfr frequency **(B)** of total $CD4^+$ T cells, separated by timepoint. **(C)** Percent change from baseline of specific Tfh subpopulations ICOS+PD1+ (left), $ICOS^{hi}CD38^{hi}$ (middle), $CD226^+Tigit^+$ (right)**,** separated by timepoint. **(D)** Percent change from baseline of different Tfh cell polarizations including Tfh1 (left), Tfh2 (middle), and Tfh17 (right) identified by indicated chemokine receptor expression. **(E)** Percent change from baseline CXCR5 (top) and PD1 (bottom) expression, measured as mean fluorescent intensity (MFI) by flow cytometry. All displayed data points represent mean value, with error bars indicating standard error. *p<0.05, uncorrected Mann-Whitney U test.

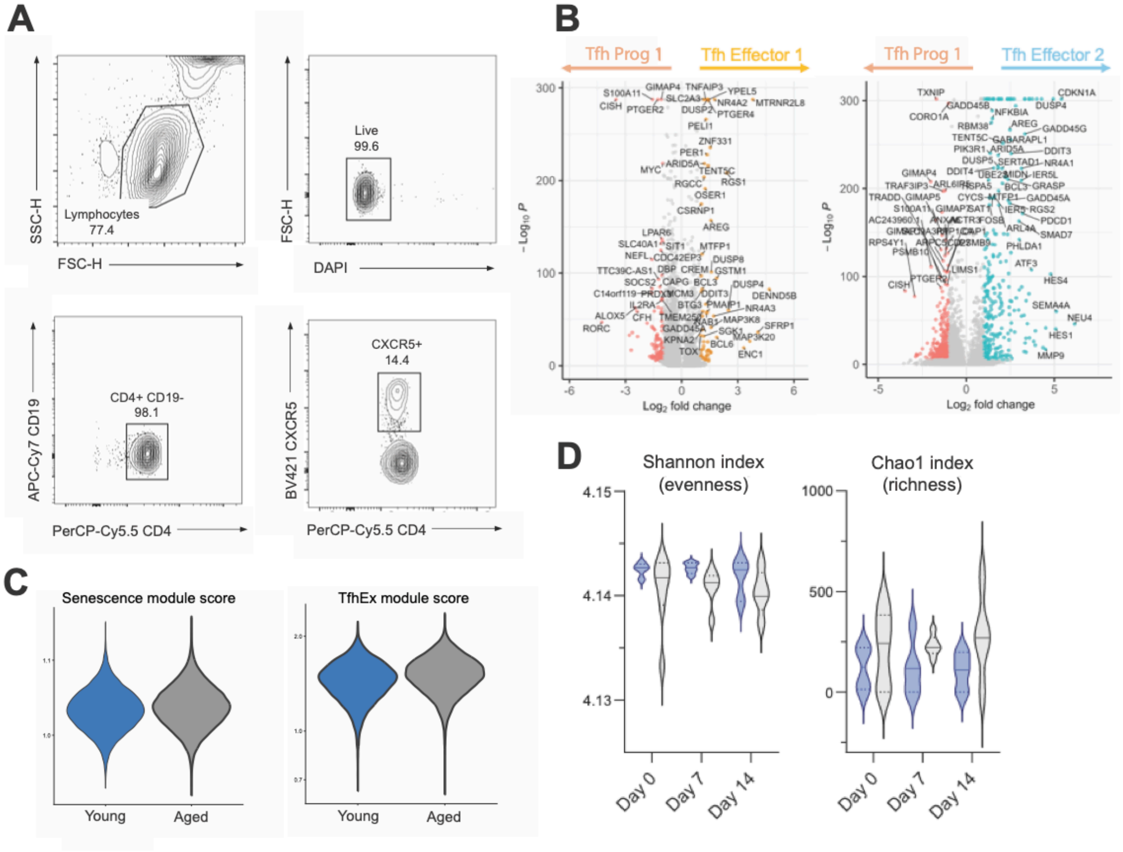

Supplemental Figure 9. Follicular T cell scRNAseq sorting strategy and additional analysis. (A) A lymphocyte gate was applied to CD4^{+}-enriched PBMCs from young and aged influenza vaccinees (upper left), the live fraction identified **(**upper right**)**, the CD4+ CD19- populations selected **(**lower left**)**, and the follicular T population identified by CXCR5 staining **(**lower right**)**. **(B)** Volcano plots summarizing differential gene expression between cells in the Tfh Naïve 1 cluster and either Tfh Effector 1 **(**left**)** or Tfh Effector 2 **(**right**)** clusters (p < 10^{-3}, FC > 1). (**C**) Violin plots of overall module score for senescence (left) and the TfhEx developmental stage (right). **(D)** Shannon **(**left**)** and Chao1 **(**right**)** indexes representing Tfh cell clonal evenness and richness/diversity (respectively) in aged (gray) and young (blue) individuals at 0, 7, and 14 days following influenza vaccination.

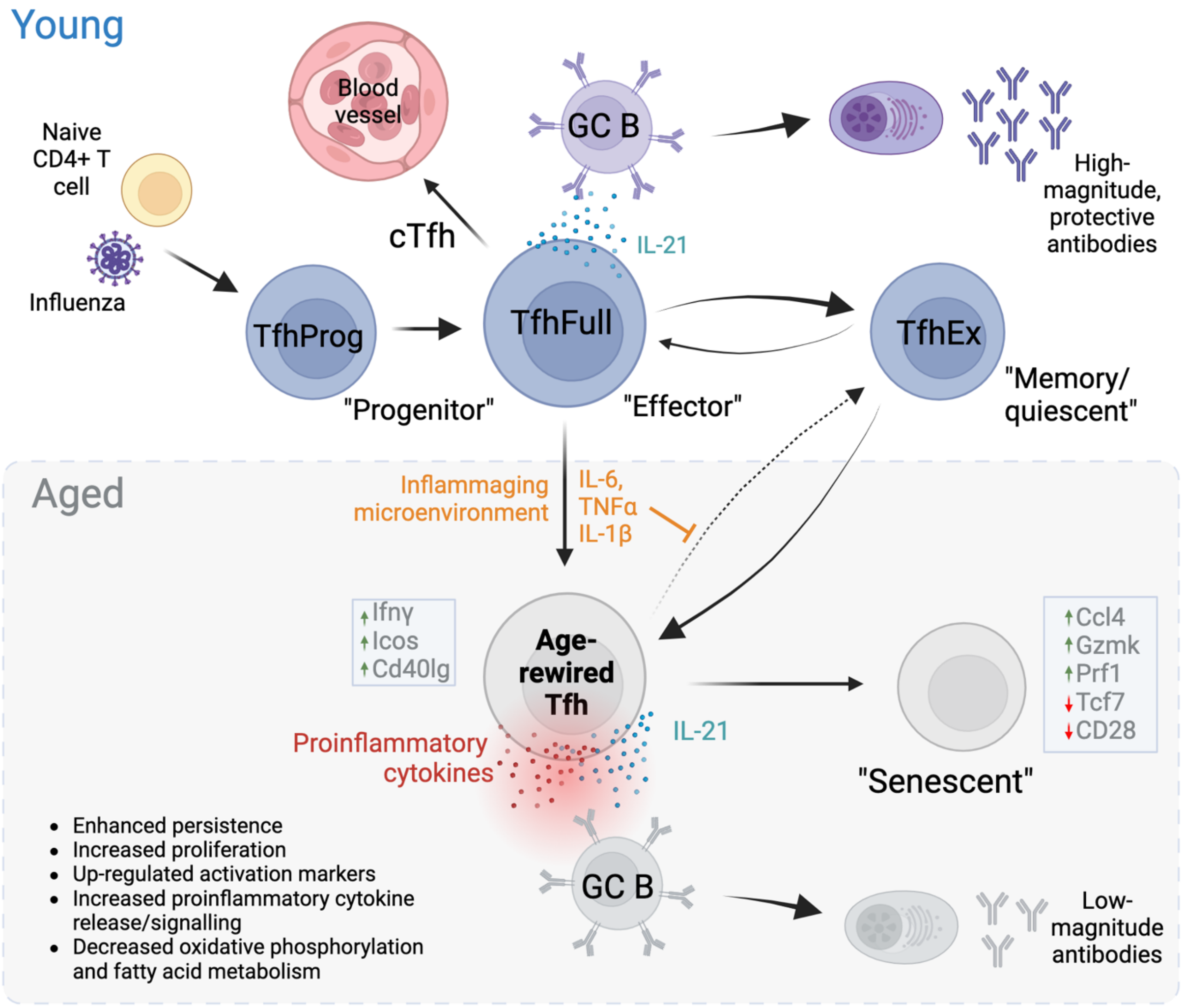

Supplemental Figure 10. Graphical abstract. Naïve CD4+ T lymphocytes differentiate into Tfh progenitor (TfhProg) and subsequently Tfh effector (TfhFull) cells upon antigen stimulation (e.g. influenza vaccination). TfhFull cells secrete IL21 and interact with germinal center B cells, stimulating them to undergo class switch recombination, affinity maturation, and to differentiate into plasma cells. This effective stimulation results in the production of a high magnitude of protective antigen-specific antibodies. A subset of TfhFull enter the bloodstream and become circulating Tfh (cTfh). TfhFull can further differentiate into the TfhEx stage which exhibits a quiescent/memory phenotype. However, when stimulated by an inflammaging microenvironment, TfhFull cells are rewired. Importantly, age-rewired Tfh cells cannot transition to the TfhEx phenotype and can produce proinflammatory cytokines such as interferon γ in abundance. When age-rewired cells interact with GC B cells, there is less effective affinity maturation and plasma cell differentiation which results in a lower magnitude of antigen-specific antibodies. Furthermore, age-rewired Tfh can subsequently differentiate into senescent Tfh which exhibit a distinct proinflammatory transcriptomic profile.

Supplemental Table 1. Mouse scRNAseq absolute cell count by age and cluster.

		Aged		Young	
Cluster	Total Cells	# Cells	%	# Cells	%
0	1340	450	33.6	890	66.4
1	1288	460	35.7	828	64.3
2	1218	660	54.2	558	45.8
3	697	158	22.7	539	77.3
4	642	563	87.7	79	12.3
5	515	175	34.0	340	66.0
6	504	153	30.4	351	69.6
7	445	173	38.9	272	61.1
8	384	341	88.8	43	11.2
9	175	53	30.3	122	69.7
10	152	151	99.3	1	0.7

Supplemental Table 2. Mouse scRNAseq senescence-associated gene differential expression.

	Gene	log2 FC	Young exp	Aged exp	Adj P
Increased in aged	Ccl4	-9.44	0	0.136	1.20E-123
	Ccl3	-7.06	0.001	0.093	5.21E-80
	Ccl5	-5.45	0.132	0.373	1.03E-159
	Spp1	-5.20	0	0.01	2.53E-05
	Esm1	-4.68	0.008	0.149	6.31E-116
	Csf1	-2.11	0.011	0.031	3.40E-05
	Icam1	-1.20	0.107	0.23	3.67E-42
	Tnf	-1.07	0.018	0.036	0.028
	Ptger2	-0.85	0.069	0.11	2.88E-06
Increased in young	Pecam1	0.55	0.307	0.214	7.10E-16
	Cd55	0.69	0.247	0.153	3.80E-19
	Il6st	0.71	0.255	0.159	3.13E-19
	Igfbp4	0.76	0.105	0.058	6.34E-09
	Ets2	0.83	0.077	0.044	3.17E-05
	Jun	1.39	0.367	0.192	3.98E-63

Supplemental Table 3. Human cohort demographic characteristics

		Aged (n=78)	Young (n=18)
Median age (range)		69 (65-84)	26 (19-38)
Sex - number (%)	Male	17 (31)	8 (44)
	Female	37 (69)	9 (56)
Vaccine type - number (%)	Standard	4 (7)	14 (88)
	High dose	42 (78)	0 (0)
	Unknown	8 (15)	2 (12)
Race - number (%)	White	50 (93)	10 (63)
	Black	0 (0)	2 (13)
	American Indian or Alaska Native	0 (0)	1 (6)
	Native Hawaiian or other Pacific Islander	0 (0)	0 (0)
	Asian	2 (4)	0 (0)
	Other, or more than one race	0 (0)	3 (19)
	Decline to answer	2 (4)	0 (0)
Ethnicity - number (%)	Hispanic or Latino	0 (0)	0 (0)
	Not Hispanic or Latino	43 (80)	15 (94)
	Decline to answer	11 (20)	1 (6)

Supplemental Table 4. Median HAI titer and MSD binding, separated by age group.

	Aged H1 HAI titer			Young H1 HAI titer			Aged H1 Binding MSD			Young H1 Binding MSD		
Timepoint	Male	Female	P*	Male	Female	P*	Male	Female	P*	Male	Female	P*
0	40	40	0.917	160	40	0.793	12461	29105	0.060	33987	26579	0.566
7	40	80	0.429	320	40	0.568	25115	30667	0.289	41472	34194	0.566
14	80	80	0.526	320	160	0.632	28117	50782	0.128	53919	43331	0.646
30	80	80	0.535	320	160	0.565	20265	34734	0.128	42279	34658	0.574

*Mann-Whitney U test, with Bonferroni p-value correction
